# Ward-specific clustering of methicillin-resistant *Staphylococcus aureus spa*-type t037 and t045 in two hospitals in South Africa: 2013 to 2017

**DOI:** 10.1371/journal.pone.0253883

**Published:** 2021-06-29

**Authors:** Wilhelmina Strasheim, Olga Perovic, Ashika Singh-Moodley, Stanford Kwanda, Arshad Ismail, Michelle Lowe

**Affiliations:** 1 Centre for Healthcare-Associated Infections, Antimicrobial Resistance and Mycoses, National Institute for Communicable Diseases, National Health Laboratory Service, Johannesburg, South Africa; 2 Faculty of Health Sciences, Department of Clinical Microbiology and Infectious Diseases, School of Pathology, University of Witwatersrand, Johannesburg, South Africa; 3 Sequencing Core Facility, National Institute for Communicable Diseases, National Health Laboratory Service, Johannesburg, South Africa; The Rockefeller University, UNITED STATES

## Abstract

**Introduction:**

Methicillin-resistant *Staphylococcus aureus* (MRSA) is a highly clonal pathogen causing infections in various settings. The aim of this study was to determine if healthcare-associated (HA) MRSA isolates with the same *spa*-type originating from two geographically distinct hospitals in South Africa were genetically related based on PFGE. Furthermore, a small subset of MRSA isolates were characterised with WGS and then compared to PFGE to determine if PFGE is still a reliable method to define outbreaks and/or transmission chains.

**Methods:**

*Staphylococcus aureus* isolated from blood cultures (BC) were submitted to the Centre for Healthcare-Associated Infections, Antimicrobial Resistance and Mycoses (CHARM) as part of a laboratory-based surveillance programme (GERMS-SA). The identified HA-MRSA isolates underwent molecular characterisation [Staphylococcal Chromosome Cassette (SCC) *mec* and *spa*-typing]. Pulsed-field gel electrophoresis (PFGE) was performed on selected isolates with the same *spa*-type. Twenty-one MRSA isolates were selected for whole-genome sequencing (WGS) based on *spa-*type, PFGE clustering, time and place of isolation.

**Results:**

Eighteen percent (n = 95/529) and 33% (n = 234/710) of isolates collected, from two public tertiary academic hospitals in the Gauteng (GAU) and the Western Cape (WC) provinces, were identified as MRSA, respectively. The most dominant clone in the GAU hospital was t037-III-MRSA (43.2%; n = 41/95). The most dominant clones in the WC hospital was t037-III-MRSA (23.9%, n = 56/234) and t045-I-MRSA (23.5%, n = 55/234). The GAU-t037-III-MRSA cases and WC-t045-I-MRSA cases occurred in the paediatric patient population, whereas the WC-t037-III-MRSA cases occurred in the adult patient population. A novel *spa*-type (t19935) was detected in the GAU hospital. PFGE showed that the GAU- and WC-t037-III-MRSA isolates were genetically indistinguishable, as well as most of the WC-t045-I-MRSA isolates. The Vienna/Hungarian/Brazilian clone and British EMRSA-3 clone were in circulation and a low frequency of single nucleotide polymorphisms (SNP) (≤20) differences was observed among isolates with the same *spa*-type.

**Conclusion:**

The low number of SNP differences is suggestive of uninterrupted strain transmission and the persistence of t037-III-MRSA and t045-I-MRSA from 2013 to 2017 in the two studied hospitals. Alternative infection prevention and control strategies should be considered to supplement control efforts.

## Introduction

Methicillin-resistant *Staphylococcus aureus* (MRSA) is listed as a high priority pathogen by the World Health Organization [1]. This bacterium can cause a wide range of infections but remains an important cause of bacteraemia in both the community and hospital setting [2]. Patients with MRSA bacteraemia pose a burden to any healthcare system, especially those with restrained resources, as these patients are hospitalised for longer, increasing their costs and are reported to have a higher risk of mortality [3,4].

MRSA was first reported in the United Kingdom (UK) in the 1960s [5]. An essential characteristic of MRSA is the presence of the *mec*A gene, harboured on the Staphylococcal Chromosome Cassette (SCC) *mec* element, which allows MRSA to survive in the presence of broad-spectrum β-lactams [6]. It was first thought that resistance emerged due to the introduction of methicillin as a therapeutic agent [7]. However, whole-genome sequencing (WGS) analysis of a historical MRSA collection showed that archaic MRSA strains were already in circulation during the 1940s and emerged due to the selective pressure imposed by antibiotic usage combined with poor infection prevention and control measures [7].

*S*. *aureus* is a highly clonal pathogen and MRSA isolates with the same Staphylococcal protein A (*spa*)*-*type suggest that these isolates were genetically related at some point in time [8,9]. However, inference of strain relatedness from an isolate’s *spa-*type is problematic, since it involves a single gene; disregards the remainder of *S*. *aureus’* genome and it may miss recombination and homoplasy events [9,10]. Pulsed-field gel electrophoresis (PFGE) has a higher discriminatory power over single gene typing schemes and when combined with epidemiological data, is a valuable tool to confirm the genetic relatedness of MRSA isolates, even though this technique is not considered as the gold standard anymore and is being replaced by WGS [9]. However, WGS is still costly in low- to middle-income countries and it is not financially feasible to sequence large historic collections of isolates.

The first reported South African cases of MRSA was in 1973 in a large paediatric hospital (2%; n = 17/843) [11]. The first hospital outbreak of MRSA in South Africa was in 1986 as nurses noted an increase in infections in the burn, plastic, orthopaedic and trauma wards [12]. Since the first reported MRSA case, an increase in the prevalence of MRSA was observed throughout the country [13–17]. The highest prevalence of MRSA was recorded in 2010 (53%; 297/556), followed by a significant decline (53% to 40%; P ≤0.001) in cases from 2012 to 2016 [17,18]. Currently, the proportion of *S*. *aureus* blood culture (BC) isolates resistant to methicillin in South Africa is ~25%, according to the Global Antimicrobial Resistance Surveillance System (GLASS) reports [19–21]. The latest GLASS report (2020) showed that the median rate observed for bloodstream related MRSA infections globally was 12.11% [interquartile range (IQR): 6.4 to 26.4], however, the report does state that the data are not nationally representative and should be interpreted with caution [21].

A previous South African study conducted from 2013 to 2016 in five public tertiary hospitals in two provinces [Gauteng (GAU) and the Western Cape (WC)] showed that the majority of MRSA bacteraemia cases were healthcare-associated (HA) [18]. The most prevalent *spa*-types identified in the studied period were t037, t045 and t1257 [18].

The aim of this study was to determine if HA-MRSA isolates (with the same *spa*-type) collected from 2013 to 2017 from two geographically distinct hospitals in South Africa were genetically related based on PFGE. Furthermore, a small subset of MRSA isolates, that were selected based on *spa-*type, PFGE clustering, time and place of isolation, were characterised with WGS and then compared to PFGE to determine if PFGE is still a reliable method to define outbreaks and/or clonal transmission. This study will shed light on the major MRSA clones in circulation as well as their genetic characteristics.

## Materials and methods

### Case definitions, study setting and ethical approval

A case of HA-*S*. *aureus* bacteraemia was defined as the isolation of *S*. *aureus* from a BC after 48 h of admission. Methicillin-resistance was defined as non-susceptibility to oxacillin [minimum inhibitory concentration (MIC) ≥ 4 μg/mL] and polymerase chain reaction (PCR) detection of the *mec*A gene. Cases originated from two public tertiary academic hospitals; one situated in the WC province and another in the GAU province. A few HA-MRSA isolates in this study originated from a previously published sentinel site surveillance study (January 2013 to January 2016) [18]. Surveillance started in the GAU hospital in 2013 (i.e. five-year period), whereas surveillance in the WC hospital started in 2014 (i.e. four-year period). The study was approved by the Human Research Ethics Committee of the University of the Witwatersrand (Protocol No: M10464) as part of an already established surveillance system (GERMS-SA) for pathogens of public health importance at the National Institute for Communicable Diseases (NICD).

### Phenotypic characterisation

Isolates were submitted on Dorset-egg transport media [Diagnostic Media Products (DMP), National Health Laboratory Service (NHLS), SA] from two regional public diagnostic laboratories to the Antimicrobial Resistance Laboratory and Culture Collection (AMRL-CC) in the Centre for Healthcare-associated infections, Antimicrobial Resistance and Mycoses (CHARM), NICD. Isolates were identified with the VITEK 2 system (bioMérieux, France) in 2013. Matrix-assisted laser desorption ionization-time of flight mass spectrometry (MALDI-TOF MS) (Microflex, Bruker Daltonics, USA) was used for identification from 2014 onwards. Antimicrobial susceptibility testing (AST) was done with the MicroScan Walkaway 96 plus system (Beckman Coulter, USA) and interpreted according to the Clinical Laboratory Standards Institute (CLSI) guidelines (2017) [22].

### Total genomic DNA extraction

Total genomic DNA was extracted using a crude boiling method. Colonies were re-suspended in 400 μL tris-ethylenediamine tetra-acetic acid (Tris-EDTA) buffer (10 mM:1mM, pH 8) and boiled at 95°C for 25 minutes, followed by centrifugation. The supernatant was stored at -20°C until used for molecular PCR testing.

### PCR detection and typing techniques

The *mec*A and the *S*. *aureus* species-specific *nuc* genes were detected on the LightCycler 480 instrument II (Roche Life Science, Germany) using previously published primer and probe sequences [23]. *S*. *aureus* ATCC 49476 was used as a positive control for the *mec*A and *nuc* PCR assay. All MRSA isolates underwent SCC*mec* typing using the multiplex PCR strategy devised by Milheiriço et al. [24,25]. The following *S*. *aureus* strains were used as positive controls for the SCC*mec* PCR assay as previously published [25]: COL (SCC*mec* I and *ccr* class 1), BK2464 (SCC*mec* II, *ccr* class 2 and *mec* class A), ANS46 (SCC*mec* III and *ccr* class 3), MW2 (SCC*mec* type IV and *mec* class B), WIS (SCC*mec* V, *ccr* class 5 and *mec* class C) and HDE 288 (SCC*mec* VI and *ccr* class 4). *Spa*-typing was done by amplifying the *sp*A locus, followed by PCR purification and Sanger sequencing [26]. Sequence assembly was done in the CLC Main Workbench (QIAGEN, Netherlands) and specific *spa*-types were assigned with the Ridom StaphType software (Ridom GmbH, Germany) [26]. Unidentified *spa*-types were submitted to the *spa-*typing website (http://www.spaserver.ridom.de/), which is curated by SeqNet.org (http://www.SeqNet.org/).

The location (GAU *vs*. WC); followed by the detected *spa*-type, the detected SCC*mec* type, methicillin-resistance and isolate number were the conventional nomenclature used to describe isolates in this study [i.e. (location)-(*spa-*type)-(SCC*mec-*type)-(MRSA)-(isolate number)].

### Pulsed-field gel electrophoresis

HA-MRSA isolates with the same *spa*-type occurring in the same hospital, in similar wards (i.e. adult and paediatric wards) were selected for PFGE. PFGE was done as previously described [27,28]. Cluster analysis was done in BioNumerics (GelCompar II Gel Electrophoresis Software, Version 7.6, Applied Math, UK). Pairwise similarity values were calculated with the band-based Dice coefficient with optimisation and a band matching tolerance set at 1.5%. Unweighted pair group matching analysis (UPGMA) was the clustering method applied. Isolates grouping with a similarity value of ≥80% were considered genetically related.

### Whole-genome sequencing and analysis

To supplement PFGE, WGS was performed on 21 selected HA-MRSA isolates to confirm the genetic relatedness. The selection was based on PFGE clustering (i.e. showed ≥ 80% sequence similarity), time (specimen collection date) and place (hospital and residing ward) of isolation for MRSA isolates with the same *spa*-type. The genomic DNA from each isolate was extracted using the QIAamp DNA Mini Kit (Qiagen, Germany) with the inclusion of lysozyme (10 mg/mL) to ensure sufficient lysis. Library preparation was done with the Nextera DNA Flex library prep kit (Illumina, USA) and sequencing was done on the MiSeq platform (Illumina, USA) at a 2x300 bp read length at a 100x coverage.

Raw sequencing reads were analysed using the Jekesa pipeline (v1.0; https://github.com/stanikae/jekesa). Briefly, Trim Galore! (v0.6.2; https://github.com/FelixKrueger/TrimGalore) was used to filter the paired-end reads (Q>30 and length >50 bp). *De novo* assembly was performed using SPAdes v3.13 [29] and the assembled contigs were polished using Shovill (v1.1.0; https://github.com/tseemann/shovill). The multilocus sequence typing (MLST) profiles were determined using the MLST tool (v2.16.4; https://github.com/tseemann/mlst). Assembly metrics were calculated using QUAST (v5.0.2; http://quast.sourceforge.net/quast). Whole-genome single nucleotide polymorphism (SNP) differences were determined with a reference-free approach using the SKA toolkit [30]. The SNP cut off value of ≤20 was used as previously published [31]. Antibiotic resistance profiles and virulence genes were predicted using ResFinder [32], PointFinder [33], VirulenceFinder [34] and NCBI AMRFinderPlus [35], implemented in the Jekesa pipeline. The Center for Genomic Epidemiology web tools (https://cge.cbs.dtu.dk/services/) were used to determine the *spa-*types, the SCC*mec* types and the incompatibility (Inc) groups of the selected MRSA isolates. Pathogenwatch (https://pathogen.watch/) was used to construct the phylogenetic tree [Newick (NWK) file]. The exported NWK file was used in Microreact (https://microreact.org/showcase) to visualise and edit the phylogenetic tree. The assembled genome files were submitted to the National Center for Biotechnology Information GenBank and are available under BioProject number: PRJNA686123.

### Statistical analysis

Data capturing was done in Microsoft Access. Statistical analysis was performed in Microsoft Excel (Microsoft, USA). Categorical variables were reported as numbers and percentages. Continuous variables were reported as means and IQR.

## Results

### Case contribution per hospital, the percentage of methicillin-resistance and phenotypic characterisation of the MRSA isolates

A total of 529 *S*. *aureus* isolates were submitted from 2013 to 2017 from the GAU hospital, whereas 710 *S*. *aureus* isolates were submitted from 2014 to 2017 from the WC hospital. Eighteen percent (n = 95/529) and 33% (n = 234/710) of *S*. *aureus* isolates were methicillin-resistant in the GAU- and the WC hospitals, respectively (Table 1). The median age of adult patients with MRSA infection in the GAU- and WC hospital was 44 years (IQR: 33–63) and 43.5 years (IQR: 30–60), respectively. The median age of paediatric patients with MRSA infection in the GAU- and WC hospital was 20 days (IQR: 10–63) and 12 days (IQR: 7–40), respectively. The number of MRSA cases detected in both adult and paediatrics patients in the GAU- and WC hospital was 14 in 2013, 76 in 2014 and 2015, 77 in 2016 and 86 in 2017. The source of bacteraemia (i.e bacteraemia without focus) could not be established for the majority of MRSA cases in the GAU and WC hospitals (n = 238/329). The antimicrobial susceptibility profiles of all MRSA isolates from the GAU- and WC hospital is also shown in Table 1.

**Table 1:** Comparison of HA-MRSA isolates from a tertiary academic hospital in the Gauteng and the Western Cape provinces (2013 to 2017).

	Patient population			
	Adults		Paediatric	
**Hospital**	**WC**	**GAU**	**WC**	**GAU**
	**% (n = 158)**	**% (n = 36)**	**% (n = 76)**	**% (n = 59)**
**Median age (IQR)**	43.5 (30–60) years	44 (33–63) years	12 (7–40) days	20 (10–63) days
**Number of cases detected per year**				
2013	0.00 (0)	25.00 (9)	0.00 (0)	8.47 (5)
2014	31.65 (50)	13.89 (5)	17.11 (13)	13.56 (8)
2015	20.25 (32)	16.67 (6)	28.95 (22)	27.12 (16)
2016	25.32 (40)	16.67 (6)	26.32 (20)	18.64 (11)
2017	22.78 (36)	27.78 (10)	27.63 (21)	32.20 (19)
**Source of BSI**				
Skin/soft tissue infection	17.09 (27)	5.56 (2)	1.32 (1)	5.08 (3)
Bacteraemia without focus	62.66 (99)	80.56 (29)	75.00 (57)	89.83 (53)
Lower respiratory tract infections	16.46 (26)	5.56 (2)	17.11 (13)	1.69 (1)
Meningitis	0.00 (0)	2.78 (1)	0.00 (0)	0.00 (0)
Joint and bone infection	1.90 (3)	2.78 (1)	2.63 (2)	0.00 (0)
Abscess	0.00 (0)	0.00 (0)	1.32 (1)	0.00 (0)
Peritonitis	1.27 (2)	0.00 (0)	0.00 (0)	0.00 (0)
Endo- and myocarditis	0.63 (1)	0.00 (0)	1.32 (1)	0.00 (0)
Unknown	0.00 (0)	2.78 (1)	1.32 (1)	3.39 (2)
**Antibiotic susceptibility profile (% R)**				
Ciprofloxacin (MIC ≥4 μg/mL)	94.94 (150)	88.89(32)	27.63 (21)	81.36 (48)
Moxifloxacin (MIC ≥2 μg/mL)	89.87 (142)	86.11 (31)	27.63 (21)	79.66 (47)
Levofloxacin (MIC ≥4 μg/mL)	94.94 (150)	86.11 (31)	23.68 (18)	81.36 (48)
Inducible clindamycin (erythromycin MIC ≥4 μg/mL and clindamycin MIC ≥0.5 μg/mL)	77.85 (123)	83.33 (30)	92.11 (70)	79.66 (47)
Mupirocin (high level ≥256 μg/mL)	5.70 (9)	5.56 (2)	6.58 (5)	5.08 (3)
Rifampicin (MIC ≥4)	18.99 (30)	47.22 (17)	7.89 (6)	25.42 (15)
Tetracycline (MIC ≥16)	51.90 (82)	55.56 (20)	14.47 (11)	81.36 (48)
Trimethoprim/Sulfamethoxazole (MIC ≥4/76)	48.72 (77)	52.78 (19)	11.84 (9)	74.58 (44)
Linezolid (MIC ≥8)	0.00 (0)	0.00 (0)	0.00 (0)	0.00 (0)
Daptomycin (No MIC breakpoint for R, S ≤1)	0.00 (0)	0.00 (0)	1.32 (1)^**^**^	0.00 (0)
Vancomycin (MIC ≥16)	0.00 (0)	0.00 (0)	0.00 (0)	0.00 (0)
***Spa*-type**				
t037	31.01 (49)	13.89 (5)	9.21 (7)	61.02 (36)
t045	3.16 (5)	8.33 (3)	65.79 (50)	13.56 (8)
t1257	15.82 (25)	44.44 (16)	5.26 (4)	10.17 (6)
t032	10.13 (16)	0.00 (0)	3.95 (3)	0.00 (0)
t012	22.78 (36)	13.89 (5)	6.58 (5)	1.69 (1)
t022	0.00 (0)	8.33 (3)	0.00 (0)	0.00 (0)
t064	0.00 (0)	0.00 (0)	0.00 (0)	3.39 (2)
Other	17.09 (27)	11.11 (4)	9.21 (7)	10.17 (6)
**SCC*mec* type [24,25]**				
I	0.00 (0)	0.00 (0)	1.32 (1)	1.69 (1)
II	27.85 (44)	16.67 (6)	7.89 (6)	1.69 (1)
III	30.38 (48)	16.67 (6)	3.95 (3)	61.02 (36)
IV	36.08 (57)	55.56 (20)	7.89 (6)	18.64 (11)
V	0.63 (1)	0.00 (0)	0.00 (0)	0.00 (0)
VI	0.63 (1)	0.00 (0)	0.00 (0)	0.00 (0)
Unknown, but *mec*A positive^**#**^	4.43 (7)	11.11 (4)	78.95 (60)	16.95 (10)
**Alternative SCC*mec* type [36]**				
SCC*mec* type I-like (1,5B)	0.63 (1)	8.33 (3)	73.68 (56)	13.56 (8)
SCC*mec* type III-like (3,5A)	0.63 (1)	0.00 (0)	0.00 (0)	0.00 (0)
Either SCC*mec* type V or SCC*mec* type VII (5C)	3.16 (5)	2.78 (1)	1.32 (1)	0.00
Polyclonal (multiple *ccr* and *mec* gene complexes)^*^	0.00 (0)	0.00 (0)	1.32 (1)	1.69 (1)
Untypeable~	0.00 (0)	0.00 (0)	2.63 (2)	1.69 (1)

GAU = Gauteng; WC = Western Cape; IQR = Interquartile range; BSI = Bloodstream infections; % R = Percentage of resistant isolates; MIC = Minimum inhibitory concentration in μg/mL.

**#** The SCC*mec* type could not be determined using the Milheiriço *et al*.[24,25] assay, but the internal control for the *mec*A gene was positive.

**^** Single isolate with MIC of 4 for daptomycin (isolate 10044).

* Polyclonal (multiple *ccr* and *mec* gene complexes): (i) Paediatric GAU—(1,5B,C), polyclonal infection can’t be excluded; (ii) Paediatric WC—polyclonal infection for a single case (1,2,5B) can’t be excluded (i.e. two SCC*mec* types were detected: SCC*mec* type IV (2B) and SCC*mec* type I-like (1,5B)).

~ Paediatric WC—two isolates were *mec*A positive but the *mec* gene complex (A, B and C) was not detected; Paediatric GAU –the *cc*r gene complex type 3 and *mec* gene complex C (3C) was detected. The structure of the SCC*mec* was not explored further.

All WC-t045-I-MRSA isolates belong to sequence type (ST) 5, clonal complex (CC) 5 and all GAU- and WC-t037-III-MRSA isolates belongs to ST239, CC8.

### *Spa*-type diversity of MRSA isolates, patient demographics of major s*pa*-types and its associated SCC*mec* types

Thirty different *spa*-types were observed in the WC hospital. *Spa-*type t037 (23.9%, n = 56/234) and t045 (23.5%, n = 55/234) were the predominant *spa*-types in circulation, followed by t012 (17.5%, n = 41/234), t1257 (12.4%, n = 29/234) and t032 (8.1%, n = 19/234). Twenty *spa*-types (t015, t0121, t223, t238, t294, t304, t324, t432, t498, t578, t1467, t2409, t2526, t5483, t5691, t6330, t6931, t11775, t11775 and t18226) were singletons. One *spa*-type could not be determined (even after multiple Sanger sequencing attempts). The unknown *spa*-type was also identified as a singleton. The remaining *spa*-types [t1971 (2.1%, n = 5/234); t1476 (1.3%, n = 3/234); t018 (0.9%, n = 2/234); t021 (0.9%, n = 2/234) and t718 (0.9%, n = 2/234)] in the WC hospital occurred at low frequencies.

Sixteen different *spa*-types were observed in the GAU hospital. The predominant *spa*-types in circulation in the GAU hospital were t037 (43.2%; n = 41/95), t1257 (23.2%, n = 22/95) and t045 (11.6%, n = 11/95). Ten *spa*-types were singletons (t008, t186, t355, t463, t718, t913, t1096, t4410, t5691 and t19935). This is the first report of the novel s*pa-*type t19935. *Spa*-type t012 (6.3%, n = 6/95), t022 (3.2%, n = 3/95) and t064 (2.1%, n = 2/95) occurred at low frequencies. *Spa-*type t037-MRSA cases in the WC hospital was associated with an adult patient population, whereas t037-MRSA cases in the GAU hospital were associated with a paediatric patient population. The SCC*mec* type III was associated with t037-MRSA cases, whereas unknown SCC*mec* elements (typed with the Milheiriço’s assay [24,25]), were associated with most of the t045-MRSA cases for both hospitals (Table 1).

### Ward distribution among *spa*-types and pulsed-field gel electrophoresis

The GAU-t1257-IV-MRSA (n = 22/22) and GAU-t045-I-MRSA (n = 11/11) cases occurred in different wards throughout the hospital. However, the GAU-t037-MRSA cases, with diverse SCC*mec* types (III/IV) occurred mostly in three paediatric wards [34.2% (n = 14/41) in neonatal ICU (NICU), 24.4% (n = 10/41) paediatric surgery ward (PSW) and 14.6% (n = 6/41) paediatric medical ICU (PMICU)]. Therefore, these 30 GAU-t037-MRSA paediatric cases, with diverse SCC*mec* types (III/IV) and three additional cases (t037-III-MRSA paediatric patients that moved between wards) were selected for PFGE. The WC-t1257-I-like/II/III/IV-MRSA (n = 29/29), WC-t032-I-like/IV-MRSA (n = 17/17) and WC-t012-II/III-like/IV-MRSA (n = 38/38) cases occurred in different adult and paediatric wards in the hospital, however, 69% (n = 38/55) of the WC-t037-II/III-MRSA cases occurred on the same floor where the adult surgical ICU (SICU) and burn unit were located. Therefore, these 38 WC-t037-II/III-MRSA adult cases were selected for PFGE.

**Fig 1:**
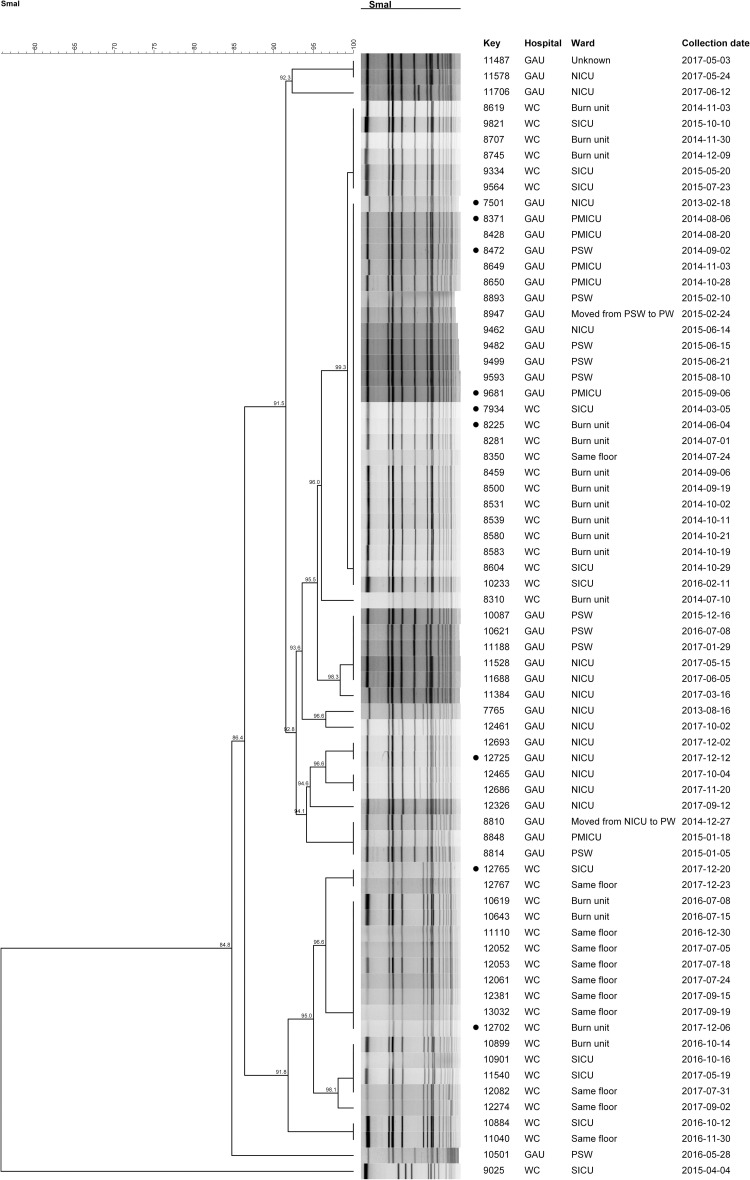
Dendrogram for t037-MRSA cases detected in the Gauteng and the Western Cape hospital. • = Isolates underwent WGS; NICU = Neonatal ICU; SICU = Surgical ICU; PMICU = Paediatric medical ICU; PSW = Paediatric surgery ward; PW = Paediatric ward; WC = Western Cape; GAU = Gauteng.

The dendrogram for both WC- and GAU-t037-MRSA cases, with diverse SCC*mec* types (II/III/IV) is shown in Fig 1. A single isolate (WC-t037-II-MRSA-9025) clustered separately from all cases [similarity value (SV) = 55.8%]. The remaining GAU- and WC-t037- MRSA cases were genetically similar (SV = 84.8%) that branched into three clusters (SV = >90%). The one group (SV = 91.8%) consisted of only WC-t037-III-MRSA cases, whereas the other group (SV = 91.5%) consisted of both WC- and GAU-t037-III-MRSA cases. Numerous WC- and GAU-t037-III-MRSA cases were genetically indistinguishable (SV = 100%).

Seventy-six percent (n = 41/54) of the WC-t045-MRSA cases occurred in paediatric wards [paediatric haematology ward (PHAE) (40.7%, n = 22/54), neonatology ward (NN) (16.7%, n = 9/54), paediatric ICU (PCIU) (11.1%, n = 6/54), neonatal high care ward (NHC) (3.7%, n = 2/54), paediatric ward (PW) (3.7%, n = 2/54)] and eight paediatric WC-t045-I-MRSA patients moved between these wards. Therefore, 49 WC-t045-I-MRSA paediatric cases were selected for PFGE.

**Fig 2:**
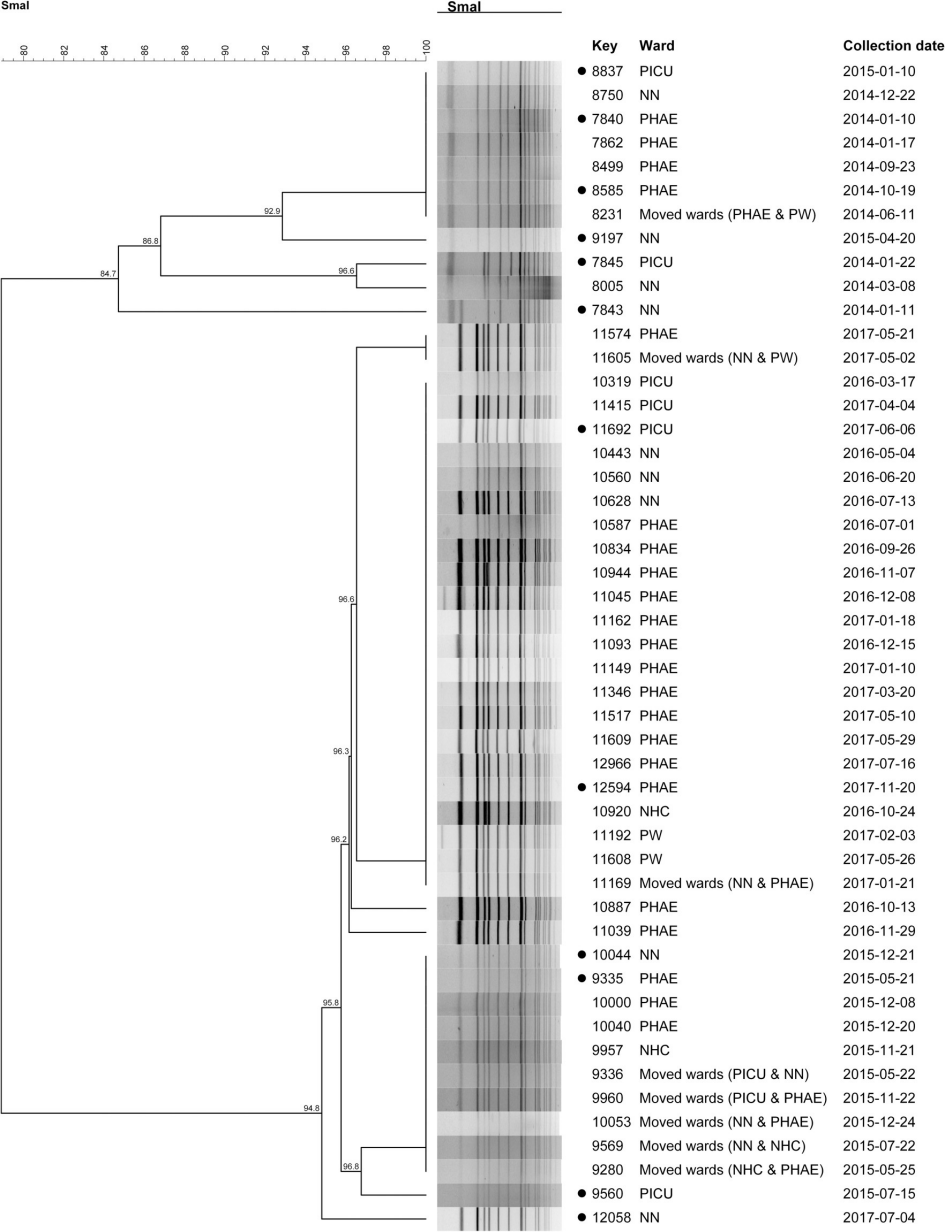
Dendrogram for t045-MRSA cases detected in the Western Cape. • = Isolates underwent WGS; NN = Neonatology, NHC = Neonatal high care; PHAE = Paediatrics haematology; PICU = Paediatric ICU; PW = Paediatric ward; WC = Western Cape.

The dendrogram for WC-t045-I-MRSA cases is shown in Fig 2. Cases grouped into two genetically similar clusters (SV = 84.7% and SV = 94.8%) and numerous cases within each cluster were genetically indistinguishable (SV = 100%).

### Whole-genome sequencing analysis

All the WC-t045-I-MRSA isolates belonged to sequence type (ST) 5 and are part of the clonal complex (CC) 5, which is also known as the pandemic British EMRSA-3 clone (ST5-I-MRSA). All the GAU- and WC-t037-III-MRSA isolates belonged to ST239 and are part of CC8, which is also known as Vienna/Hungarian/Brazilian clone (ST239-III-MRSA). The *spa*-type and SCC*mec* type data were in accordance with the WGS results.

The GAU- and WC-t037-III-MRSA harboured the RepA_N (*rep*20; n = 9/9), Rep1 (*rep*21; n = 9/9); Rep_trans (*rep*7a; n = 8/9); RepL (*rep*10; n = 2/9) and Rep3 (*rep*5a; n = 1/9) Inc groups. The WC-t045-I-MRSA isolates harboured the Rep1 (*rep*21; n = 12/12) and RepL (*rep*10; n = 4/12) Inc groups. All GAU- and WC-t037-III-MRSA isolates as well as the WC-t045-I-MRSA isolates harboured the Rep1 (*rep*21; n = 21/21).

**Fig 3:**
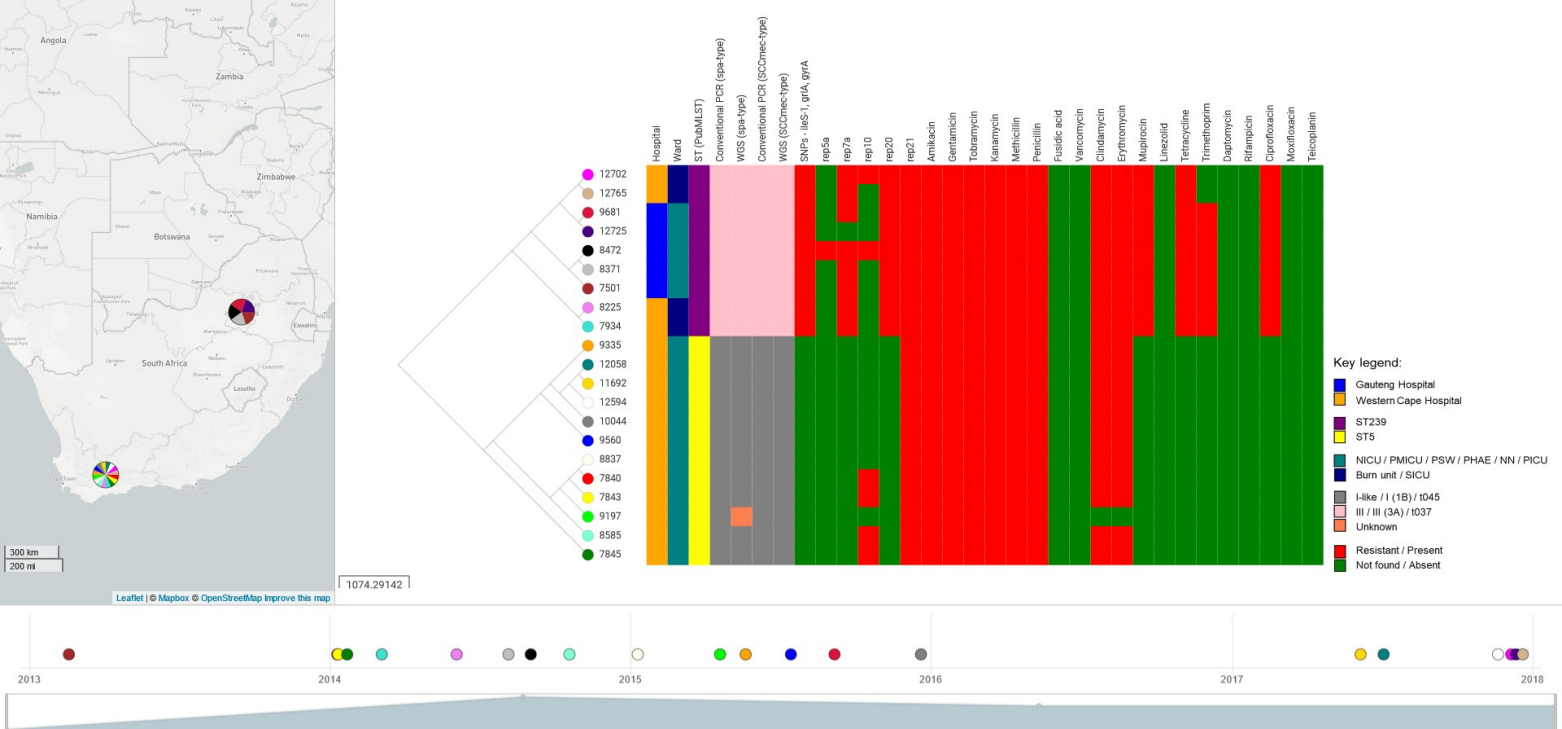
Molecular and phylogenetic comparison of a selected set of MRSA strains (n = 21). The phylogenetic tree and timeline was drawn with Microreact version 92.0.0 (https://microreact.org/project/sbBQtaWKnKdCHZ6L7bbQgJ/a639f664). NICU = Neonatal ICU; PMICU = Paediatric medical ICU; PSW = Paediatric surgery ward; PHAE = Paediatrics haematology; NN = Neonatology; PICU = Paediatric ICU; SICU = Surgical ICU. Reprinted from https://microreact.org/project/sbBQtaWKnKdCHZ6L7bbQgJ/a639f664 under a CC BY licence, with permission from the Centre for Genomic Pathogen Surveillance.

All MRSA isolates that underwent WGS (n = 21/21) were susceptible to fusidic acid, vancomycin, daptomycin, rifampicin, moxifloxacin and teicoplanin but were resistant to all aminoglycosides (amikacin, gentamicin, tobramycin, kanamycin) (Fig 3). All MRSA isolates (n = 21/21) contained the *aac*A-*aph*D (gentamicin, tobramycin and kanamycin) and *mec*A (methicillin and penicillin) antibiotic resistance genes as well as the haemolysins (*hlg*A, *hlg*B and *hlg*C genes), leukocidins (*luk*D *and luk*E genes), serine proteases (*spl*A and *spl*B) and aureolysin (*aur* gene) virulence genes. All GAU- and WC-t037-III-MRSA isolates contained the following SNPs: (i) *ile*S-1 (V588F); (ii) *grl*A (S80F) and (iii) *gyr*A (S84L). These SNPs gave rise to mupirocin and ciprofloxacin resistance (Fig 3).

The GAU-t037-III-MRSA-7501 (isolated in 2013—first isolate collected) and GAU-t037-III-MRSA-12725 (isolated in 2017—the last isolate collected) had 211 SNP differences, whereas the WC-t045-I-MRSA-7840 (isolated in 2014—first isolate collected) and WC-t037-III-MRSA-12765 (isolated in 2017—the last isolate collected) had 11296 SNP differences. The SNP differences are provided in S1 and S2 Tables.

The SNP differences (0 to 18) between GAU- and WC-t037-III-MRSA isolates as well as WC-t045-I-MRSA isolates are shown in S3 Table. It is evident that the same GAU-t037-III-MRSA clone is circulating between the PMICU and PSW, the WC-t037-III-MRSA clone is circulating between the burn unit and the SICU ward and the WC-t045-I-MRSA clone is circulating between PHAE, PICU and NN wards.

## Discussion

We investigated the genetic relatedness of HA-associated MRSA isolates at two hospitals in South Africa; as the MRSA cases shared the same *spa*-type and occurred in adult and paediatric wards in the respective hospitals. PFGE and WGS were used to genetically characterise the MRSA isolates.

PFGE showed that t037-III-MRSA and t045-I-MRSA isolates were genetically indistinguishable. PFGE and WGS results were mostly comparable and PFGE can still be used as a reliable method to determine transmission chains and outbreaks in low- to middle-income countries. However, WGS is recommended, as it provides more information on the genetic diversity of the isolates.

WGS showed that all the identified plasmids in this study only carried one *rep* gene sequence. *S*. *aureus* plasmids that carry more than one *rep* gene sequence has been identified previously [37,38]. There is currently no clear understanding of how virulence- and resistance genes are linked to *rep* genes and plasmids in *S*. *aureus* [37]. The most dominant *rep* genes detected in this study was *rep*20 (carried on the RepA_N Inc group), *rep*21 (carried on the Rep1 Inc group) and *rep*7a (carried on the Rep_trans Inc group). *Rep*5, *rep*7, *rep*10, *rep*20 and r*ep*21 was also described in human, animal and food *S*. *aureus* isolates of which *rep*10 and *rep*21 was the most dominant *rep* gene sequences [39]. The previously mentioned *rep* genes except for *rep*5 were also detected in a study conducted by Strommenger et al. in 174 *S*. *aureus* isolates from 33 different countries on five continents from 1957 to 2008 [38].

Two pandemic clones [i.e. Vienna/Hungarian/Brazilian clone (ST239-MRSA-III) and British EMRSA-3 clone (ST5-MRSA-I)] were in circulation in the two studied hospitals and a low frequency of SNP differences (SNPs ≤20) was observed among isolates with the same *spa*-type occurring in adult and paediatric wards. A definite epidemiological SNP cut-off value to determine strain relatedness in MRSA is not yet established, but low core genome SNP values (≤20) are suggestive of relatedness and therefore a recent transmission event originating from the same source [31,40,41]. A study by Harris et al. [40] estimated that one core SNP mutation occurs roughly every six weeks, which equates to the accumulation of roughly eight to nine SNP differences per year within MRSA’s genome [40]. A study by Ankrum and Hall [41] classified *S*. *aureus* strains as the same if ≤71 SNP differences were observed. Another study by Goyal et al. [31] recommended a median cut-off value of 20 SNP differences [31].

The low frequency of SNPs observed among: i) paediatric GAU-t037-III-MRSA isolates, ii) among the surgical and burn adult WC-t037-III-MRSA isolates and iii) among paediatric WC-t045-I-MRSA isolates, are all strongly suggestive that the same strain has been in circulation throughout the study period (i.e. 2013/2014 to 2017). There is some evidence in the literature that t045-MRSA-I and t037-MRSA-III strains have been in circulation for an even longer period, as a study by Moodley et al. [42] reported the circulation of ST5-t045-MRSA-I isolates in the WC and ST239-t037-MRSA-III isolates in GAU, from a set of 320 MRSA isolates originating from various clinical specimens collected from 2005 to 2006 across public and private laboratories in South Africa.

The uninterrupted transmission of the same strain type indicates a lapse in infection prevention and control, which is potentially suggestive of asymptomatic carriage by healthcare workers (HCWs). MRSA can survive on dry surfaces for an extended period and can contaminate the hands of HCW from where it can be transmitted to patients [43,44]. Another potential reason for the uninterrupted transmission is overcrowding of hospital wards and staff shortages. In South Africa, a survey of public medical and surgical unit nurses showed that the nurse-to-patient ratio ranged between 1:8.75 and 1:32 [45], whereas a national audit showed that there are 0.3 trained ICU nurses per ICU/high-care bed [46]. The adoption of routine screening of HCWs as part of occupational health and safety, followed by decolonisation, as well as deep environmental cleaning and hand hygiene compliance, to eradicate MRSA is recommended [43,44,47]. The implementation of such policy measures will disrupt the continuous transmission of the same strain and will lead to a decreased prevalence of MRSA. However, routine screening of HCWs are part of policies in high-income countries and implementation in a resource-poor setting might pose additional challenges, as HCWs colonised with MRSA will need to be placed on paid sick leave until successfully decolonised and might place additional pressure on an already short-staffed unit [47].

The study has some limitations. The study was retrospective and a secondary analysis of a previously reported surveillance programme. The definite source of MRSA could not be established as the genetic relatedness between the cases were detected after the completion of the surveillance period. In addition, only MRSA isolates underwent *spa*-typing and some transmission events involving MSSA could have been missed. Also, the presence of the Panton–Valentine leukocidin (*pvl*) gene in the study isolates was not investigated as it was not part of the aim of the study.

## Supporting information

S1 Table SNP differences between MRSA-t037 isolates submitted for WGS. (DOCX)

S2 Table SNP differences between MRSA-t045 isolates submitted for WGS. (DOCX)

S3 Table SNP differences between t037-III and t045-I-MRSA isolates (SNP cut off value ≤20 SNP differences). (DOCX)

S4 Table MRSA dataset. (DOCX)
