# Supplementary material for: Ward-specific clustering of methicillin-resistant Staphylococcus aureus spa-type t037 and t045 in two hospitals in South Africa: 2013 to 2017
Source: PLoS One. 2021 Jun 29;16(6):e0253883. doi: 10.1371/journal.pone.0253883 (PMC8241065; doi:10.1371/journal.pone.0253883)
Supplement: S1 Table — (DOCX) [file pone.0253883.s001.docx]

# Supporting information

**S1 Table. SNP differences between MRSA-t037 isolates submitted for WGS**

| ID [1] | ID [2] | SNP differences |
| --- | --- | --- |
| 7501 | 7934 | 170 |
|  | 8225 | 169 |
|  | 8371 | 40 |
|  | 8472 | 37 |
|  | 9681 | 169 |
| 7934 | 8225 | 1 |
|  | 8371 | 186 |
|  | 8472 | 183 |
|  | 9681 | 198 |
| 8225 | 8371 | 187 |
|  | 8472 | 184 |
|  | 9681 | 199 |
| 8371 | 8472 | 5 |
|  | 9681 | 302 |
| 8472 | 8585 | 11506 |
|  | 9681 | 299 |
| 12702 | 12725 | 218 |
|  | 7501 | 172 |
|  | 7934 | 204 |
|  | 8225 | 205 |
|  | 8371 | 190 |
|  | 8472 | 187 |
|  | 12765 | 8 |
|  | 9681 | 173 |
| 12725 | 12765 | 224 |
|  | 7501 | 211 |
|  | 7934 | 241 |
|  | 8225 | 242 |
|  | 8371 | 340 |
|  | 8472 | 337 |
|  | 9681 | 98 |
| 12765 | 7501 | 182 |
|  | 7934 | 210 |
|  | 8225 | 211 |
|  | 8371 | 196 |
|  | 8472 | 193 |
|  | 9681 | 179 |
