# Supplementary material for: Ward-specific clustering of methicillin-resistant Staphylococcus aureus spa-type t037 and t045 in two hospitals in South Africa: 2013 to 2017
Source: PLoS One. 2021 Jun 29;16(6):e0253883. doi: 10.1371/journal.pone.0253883 (PMC8241065; doi:10.1371/journal.pone.0253883)
Supplement: S2 Table — (DOCX) [file pone.0253883.s002.docx]

# Supporting information

**S2 Table. SNP differences between MRSA-t045 isolates submitted for WGS**

| **ID [1]** | **ID [2]** | **SNP differences** |
| --- | --- | --- |
| 7840 | 7843 | 0 |
|  | 8837 | 5 |
|  | 8585 | 6 |
|  | 9197 | 6 |
|  | 7845 | 8 |
|  | 9335 | 415 |
|  | 9560 | 415 |
| 7843 | 8837 | 5 |
|  | 8585 | 6 |
|  | 9197 | 6 |
|  | 7845 | 8 |
|  | 9335 | 415 |
|  | 9560 | 415 |
| 7845 | 9197 | 11 |
|  | 8837 | 12 |
|  | 8585 | 13 |
|  | 9335 | 410 |
|  | 9560 | 411 |
| 8585 | 8837 | 9 |
|  | 9197 | 12 |
|  | 9335 | 421 |
|  | 9560 | 421 |
| 8837 | 9197 | 11 |
|  | 9335 | 420 |
|  | 9560 | 420 |
| 9197 | 9335 | 416 |
|  | 9560 | 416 |
| 9335 | 9560 | 4 |
| 10044 | 12594 | 21 |
|  | 7840 | 420 |
|  | 7843 | 420 |
|  | 7845 | 415 |
|  | 8585 | 426 |
|  | 8837 | 425 |
|  | 9197 | 421 |
|  | 9335 | 4 |
|  | 9560 | 8 |
|  | 11692 | 14 |
|  | 12058 | 17 |
| 11692 | 12058 | 3 |
|  | 7840 | 425 |
|  | 7843 | 425 |
|  | 7845 | 420 |
|  | 8585 | 431 |
|  | 8837 | 430 |
|  | 9197 | 425 |
|  | 9335 | 10 |
|  | 9560 | 14 |
|  | 12594 | 15 |
| 12058 | 7840 | 428 |
|  | 7843 | 428 |
|  | 7845 | 423 |
|  | 8585 | 434 |
|  | 8837 | 433 |
|  | 9197 | 429 |
|  | 9335 | 13 |
|  | 9560 | 17 |
|  | 12594 | 18 |

**S2 Table. SNP differences between t045 isolates submitted for WGS (continued)**

| **ID [1]** | **ID [2]** | **SNP differences** |
| --- | --- | --- |
| 12594 | 12765 | 11359 |
|  | 7840 | 432 |
|  | 7843 | 432 |
|  | 7845 | 427 |
|  | 8585 | 438 |
|  | 8837 | 437 |
|  | 9197 | 433 |
|  | 9560 | 21 |
|  | 9335 | 17 |
