# Supplementary material for: Ward-specific clustering of methicillin-resistant Staphylococcus aureus spa-type t037 and t045 in two hospitals in South Africa: 2013 to 2017
Source: PLoS One. 2021 Jun 29;16(6):e0253883. doi: 10.1371/journal.pone.0253883 (PMC8241065; doi:10.1371/journal.pone.0253883)
Supplement: S3 Table — (DOCX) [file pone.0253883.s003.docx]

# Supporting information

**S3 Table: SNP differences between t037-III and t045-I-MRSA isolates (cut off value ≤20 SNP differences)**

| **ID [1]** | **Province, *spa*- and SCC*mec* type** | **Ward** | **Isolation date (dd/mm/yyyy)** | **ID [2]** | **Province, *spa*- and SCC*mec* type** | **Ward** | **Isolation date (dd/mm/yyyy)** | **SNP differences** |
| --- | --- | --- | --- | --- | --- | --- | --- | --- |
| **7840** | WC-t045-I-MRSA | PHAE | 10/01/2014 | **7843** | WC-t045-I-MRSA | NN | 11/01/2014 | 0 |
| **7934** | WC-t037-III-MRSA | SICU | 05/03/2014 | **8225** | WC-t037-III-MRSA | BURN UNIT | 04/06/2014 | 1 |
| **11692** | WC-t045-I-MRSA | PICU | 06/06/2017 | **12058** | WC-t045-I-MRSA | NN | 04/07/2017 | 3 |
| **9335** | WC-t045-I-MRSA | PHAE | 21/05/2015 | **9560** | WC-t045-I-MRSA | PICU | 15/07/2015 | 4 |
| **10044** | WC-t045-I-MRSA | NN | 20/12/2015 | **9335** | WC-t045-I-MRSA | PHAE | 21/05/2015 | 4 |
| **7840** | WC-t045-I-MRSA | PHAE | 10/01/2014 | **8837** | WC-t045-I-MRSA | PICU | 10/01/2015 | 5 |
| **7843** | WC-t045-I-MRSA | NN | 11/01/2014 | **8837** | WC-t045-I-MRSA | PICU | 10/01/2015 | 5 |
| **8371** | GAU-t037-III-MRSA | PMICU | 06/08/2014 | **8472** | GAU-t037-III-MRSA | PSW | 02/09/2014 | 5 |
| **7840** | WC-t045-I-MRSA | PHAE | 10/01/2014 | **8585** | WC-t045-I-MRSA | PHAE | 19/10/2014 | 6 |
| **7840** | WC-t045-I-MRSA | PHAE | 10/01/2014 | **9197** | WC-Unknown-I-MRSA* | NN | 20/04/2015 | 6 |
| **7843** | WC-t045-I-MRSA | NN | 11/01/2014 | **8585** | WC-t045-I-MRSA | PHAE | 19/10/2014 | 6 |
| **7843** | WC-t045-I-MRSA | NN | 11/01/2014 | **9197** | WC-Unknown-I-MRSA* | NN | 20/04/2015 | 6 |
| **7840** | WC-t045-I-MRSA | PHAE | 10/01/2014 | **7845** | WC-t045-I-MRSA | PICU | 22/01/2014 | 8 |
| **7843** | WC-t045-I-MRSA | NN | 11/01/2014 | **7845** | WC-t045-I-MRSA | PICU | 22/01/2014 | 8 |
| **10044** | WC-t045-I-MRSA | NN | 20/12/2015 | **9560** | WC-t045-I-MRSA | PICU | 15/07/2015 | 8 |
| **12702** | WC-t037-III-MRSA | BURN UNIT | 06/12/2017 | **12765** | WC-t037-III-MRSA | SICU | 20/12/2017 | 8 |
| **8585** | WC-t045-I-MRSA | PHAE | 19/10/2014 | **8837** | WC-t045-I-MRSA | PICU | 10/01/2015 | 9 |
| **11692** | WC-t045-I-MRSA | PICU | 06/06/2017 | **9335** | WC-t045-I-MRSA | PHAE | 21/05/2015 | 10 |
| **7845** | WC-t045-I-MRSA | PICU | 22/01/2014 | **9197** | WC-Unknown-I-MRSA* | NN | 20/04/2015 | 11 |
| **8837** | WC-t045-I-MRSA | PICU | 10/01/2015 | **9197** | WC-Unknown-I-MRSA* | NN | 20/04/2015 | 11 |
| **7845** | WC-t045-I-MRSA | PICU | 22/01/2014 | **8837** | WC-t045-I-MRSA | PICU | 10/01/2015 | 12 |
| **8585** | WC-t045-I-MRSA | PHAE | 19/10/2014 | **9197** | WC-Unknown-I-MRSA* | NN | 20/04/2015 | 12 |
| **7845** | WC-t045-I-MRSA | PICU | 22/01/2014 | **8585** | WC-t045-I-MRSA | PHAE | 19/10/2014 | 13 |
| **12058** | WC-t045-I-MRSA | NN | 04/07/2017 | **9335** | WC-t045-I-MRSA | PHAE | 21/05/2015 | 13 |
| **10044** | WC-t045-I-MRSA | NN | 20/12/2015 | **11692** | WC-t045-I-MRSA | PICU | 06/06/2017 | 14 |
| **11692** | WC-t045-I-MRSA | PICU | 06/06/2017 | **9560** | WC-t045-I-MRSA | PICU | 15/07/2015 | 14 |
| **11692** | WC-t045-I-MRSA | PICU | 06/06/2017 | **12594** | WC-t045-I-MRSA | PHAE | 20/11/2017 | 15 |
| **10044** | WC-t045-I-MRSA | NN | 20/12/2015 | **12058** | WC-t045-I-MRSA | NN | 04/07/2017 | 17 |
| **12058** | WC-t045-I-MRSA | NN | 04/07/2017 | **9560** | WC-t045-I-MRSA | PICU | 15/07/2015 | 17 |
| **12594** | WC-t045-I-MRSA | PHAE | 20/11/2017 | **9335** | WC-t045-I-MRSA | PHAE | 21/05/2015 | 17 |
| **12058** | WC-t045-I-MRSA | NN | 04/07/2017 | **12594** | WC-t045-I-MRSA | PHAE | 20/11/2017 | 18 |
| SNP = Single nucleotide polymorphism/s; SICU = Surgical ICU; NN = Neonatology; PHAE = Paediatrics haematology; PICU = Paediatric ICU; PMICU = Paediatric medical ICU; PSW = Paediatric surgery ward; WC = Western Cape; GAU = Gauteng; * The *spa*-type of isolate 9197 could not be determined with WGS but with conventional PCR the *spa*-type was identified as t045. | | | | | | | | |
