# Supplementary material for: Ward-specific clustering of methicillin-resistant Staphylococcus aureus spa-type t037 and t045 in two hospitals in South Africa: 2013 to 2017
Source: PLoS One. 2021 Jun 29;16(6):e0253883. doi: 10.1371/journal.pone.0253883 (PMC8241065; doi:10.1371/journal.pone.0253883)
Supplement: S4 Table — (DOCX) [file pone.0253883.s004.docx]

# Supporting information

**S4 Table: MRSA dataset**

| **Laboratory number** | **Year** | **Province** | **Ward** | **Age** | **Age units** | **Specimen type** | **Source of infection** | **SCC*mec* type** | ***mec*A PCR** | ***Spa*-type** | **Organism** |
| --- | --- | --- | --- | --- | --- | --- | --- | --- | --- | --- | --- |
| 7485 | 2013 | GAU | ADULT | 47 | Y | Blood culture | Unknown | IV | Positive | t022 | MRSA |
| 7501 | 2013 | GAU | PAED | 8 | D | Blood culture | Bacteraemia without focus | III | Positive | t037 | MRSA |
| 7503 | 2013 | GAU | ADULT | 31 | Y | Blood culture | Bacteraemia without focus | SCCmec type I-like (1,5B) | Positive | t045 | MRSA |
| 7547 | 2013 | GAU | ADULT | 17 | Y | Blood culture | Bacteraemia without focus | III | Positive | t037 | MRSA |
| 7605 | 2013 | GAU | ADULT | 33 | Y | Blood culture | Bacteraemia without focus | IV | Positive | t1257 | MRSA |
| 7695 | 2013 | GAU | ADULT | 56 | Y | Blood culture | Bacteraemia without focus | IV | Positive | t1257 | MRSA |
| 7632 | 2013 | GAU | PAED | 32 | M | Blood culture | Bacteraemia without focus | III | Positive | t037 | MRSA |
| 7627 | 2013 | GAU | ADULT | 34 | Y | Blood culture | Bacteraemia without focus | IV | Positive | t022 | MRSA |
| 7745 | 2013 | GAU | ADULT | 69 | Y | Blood culture | Bacteraemia without focus | IV | Positive | t1257 | MRSA |
| 7749 | 2013 | GAU | ADULT | 38 | Y | Blood culture | Bacteraemia without focus | IV | Positive | t1257 | MRSA |
| 7730 | 2013 | GAU | PAED | 12 | D | Blood culture | Bacteraemia without focus | IV | Positive | t064 | MRSA |
| 7759 | 2013 | GAU | ADULT | 44 | Y | Blood culture | Bacteraemia without focus | IV | Positive | t1257 | MRSA |
| 7765 | 2013 | GAU | PAED | 8 | D | Blood culture | Bacteraemia without focus | III | Positive | t037 | MRSA |
| 7775 | 2013 | GAU | PAED | 51 | M | Blood culture | Bacteraemia without focus | IV | Positive | t064 | MRSA |
| 7937 | 2014 | GAU | PAED | 4 | M | Blood culture | Bacteraemia without focus | IV | Positive | t1257 | MRSA |
| 8089 | 2014 | GAU | ADULT | 54 | Y | Blood culture | Bacteraemia without focus | IV | Positive | t1257 | MRSA |
| 8137 | 2014 | GAU | ADULT | 37 | Y | Blood culture | Bacteraemia without focus | II | Positive | t913 | MRSA |
| 8227 | 2014 | GAU | ADULT | 75 | Y | Blood culture | Bacteraemia without focus | IV | Positive | t008 | MRSA |
| 8371 | 2014 | GAU | PAED | 3 | D | Blood culture | Bacteraemia without focus | IV | Positive | t037 | MRSA |
| 8428 | 2014 | GAU | PAED | 16 | D | Blood culture | Skin/soft tissue infection | III | Positive | t037 | MRSA |
| 8432 | 2014 | GAU | ADULT | 30 | Y | Blood culture | Bacteraemia without focus | III | Positive | t4410 | MRSA |
| 8472 | 2014 | GAU | PAED | 11 | D | Blood culture | Bacteraemia without focus | III | Positive | t037 | MRSA |
| 8478 | 2014 | GAU | ADULT | 68 | Y | Blood culture | Bacteraemia without focus | II | Positive | t012 | MRSA |
| 8649 | 2014 | GAU | PAED | 19 | M | Blood culture | Skin/soft tissue infection | III | Positive | t037 | MRSA |
| 8650 | 2014 | GAU | PAED | 18 | D | Blood culture | Skin/soft tissue infection | III | Positive | t037 | MRSA |
| 8709 | 2014 | GAU | PAED | 1 | M | Blood culture | Bacteraemia without focus | SCCmec type I-like (1,5B) | Positive | t045 | MRSA |
| 8810 | 2014 | GAU | PAED | 4 | D | Blood culture | Bacteraemia without focus | III | Positive | t037 | MRSA |
| 8814 | 2015 | GAU | PAED | 2 | M | Blood culture | Bacteraemia without focus | III | Positive | t037 | MRSA |
| 8848 | 2015 | GAU | PAED | 1 | M | Blood culture | Bacteraemia without focus | III | Positive | t037 | MRSA |
| 8893 | 2015 | GAU | PAED | 21 | D | Blood culture | Bacteraemia without focus | III | Positive | t037 | MRSA |
| 8947 | 2015 | GAU | PAED | 3 | M | Blood culture | Bacteraemia without focus | III | Positive | t037 | MRSA |
| 9036 | 2015 | GAU | PAED | 1 | M | Blood culture | Bacteraemia without focus | IV | Positive | t037 | MRSA |
| 9254 | 2015 | GAU | ADULT | 33 | Y | Blood culture | Bacteraemia without focus | IV | Positive | t1257 | MRSA |
| 9427 | 2015 | GAU | PAED | 17 | D | Blood culture | Bacteraemia without focus | IV | Positive | t1257 | MRSA |
| 9462 | 2015 | GAU | PAED | 16 | D | Blood culture | Bacteraemia without focus | III | Positive | t037 | MRSA |
| 9482 | 2015 | GAU | PAED | 1 | M | Blood culture | Bacteraemia without focus | III | Positive | t037 | MRSA |
| 9485 | 2015 | GAU | PAED | 3 | D | Blood culture | Bacteraemia without focus | III | Positive | t355 | MRSA |
| 9567 | 2015 | GAU | ADULT | 44 | Y | Blood culture | Bacteraemia without focus | IV | Positive | t1257 | MRSA |
| 9593 | 2015 | GAU | PAED | 6 | D | Blood culture | Bacteraemia without focus | III | Positive | t037 | MRSA |
| 9615 | 2015 | GAU | PAED | 8 | M | Blood culture | Bacteraemia without focus | SCCmec type I-like (1,5B) | Positive | t045 | MRSA |
| 9617 | 2015 | GAU | ADULT | 75 | Y | Blood culture | Joint infection | IV | Positive | t1257 | MRSA |
| 9681 | 2015 | GAU | PAED | 4 | M | Blood culture | Bacteraemia without focus | III | Positive | t037 | MRSA |
| 9686 | 2015 | GAU | PAED | 15 | Y | Blood culture | Bacteraemia without focus | I | Positive | t186 | MRSA |
| 9832 | 2015 | GAU | ADULT | 26 | Y | Blood culture | Bacteraemia without focus | SCCmec type I-like (1,5B) | Positive | t463 | MRSA |
| 9997 | 2015 | GAU | ADULT | 19 | Y | Blood culture | Bacteraemia without focus | IV | Positive | t1257 | MRSA |
| 10087 | 2015 | GAU | PAED | 7 | D | Blood culture | Bacteraemia without focus | III | Positive | t037 | MRSA |
| 10111 | 2016 | GAU | PAED | 19 | D | Blood culture | Bacteraemia without focus | IV | Positive | t1257 | MRSA |
| 10116 | 2016 | GAU | PAED | 10 | Y | Blood culture | LRTI | IV | Positive | t1257 | MRSA |
| 10239 | 2016 | GAU | PAED | 4 | D | Blood culture | Bacteraemia without focus | SCCmec type-I like (1,5B) | Positive | t045 | MRSA |
| 10302 | 2016 | GAU | ADULT | 73 | Y | Blood culture | Bacteraemia without focus | III | Positive | t037 | MRSA |
| 10307 | 2016 | GAU | ADULT | 58 | Y | Blood culture | Bacteraemia without focus | IV | Positive | t1257 | MRSA |
| 10295 | 2016 | GAU | PAED | 22 | D | Blood culture | Bacteraemia without focus | SCCmec type inconclusive (1,5BC). Polyclonal infection not excluded. | Positive | t045 | MRSA |
| 10298 | 2016 | GAU | PAED | 24 | D | Blood culture | Bacteraemia without focus | Mec gene complex could not be amplified. Ccr gene complex 1 and 5 positive. Based on spa-type suggestive of SCCmec type I-like, but need to be confirmed with sequencing of complete SCCmec element | Positive | t045 | MRSA |
| 10438 | 2016 | GAU | ADULT | 73 | Y | Blood culture | Bacteraemia without focus | III | Positive | t037 | MRSA |
| 10454 | 2016 | GAU | PAED | 15 | Y | Blood culture | Bacteraemia without focus | II | Positive | t012 | MRSA |
| 10501 | 2016 | GAU | PAED | 1 | M | Blood culture | Bacteraemia without focus | III | Positive | t037 | MRSA |
| 11094 | 2016 | GAU | PAED | 29 | D | Blood culture | Bacteraemia without focus | SCCmec type-I like (1,5B) | Positive | t045 | MRSA |
| 11105 | 2016 | GAU | PAED | 7 | D | Blood culture | Bacteraemia without focus | IV | Positive | t1257 | MRSA |
| 12262 | 2017 | GAU | PAED | 20 | D | Blood culture | Bacteraemia without focus | SCCmec type-I like (1,5B) | Positive | t1096 | MRSA |
| 9026 | 2015 | GAU | PAED | 14 | M | Blood culture | Bacteraemia without focus | III | Positive | t037 | MRSA |
| 9499 | 2015 | GAU | PAED | 13 | D | Blood culture | Bacteraemia without focus | III | Positive | t037 | MRSA |
| 9603 | 2015 | GAU | ADULT | 44 | Y | Blood culture | Bacteraemia without focus | IV | Positive | t1257 | MRSA |
| 10294 | 2016 | GAU | ADULT | 18 | Y | Blood culture | Meningitis | IV | Positive | t1257 | MRSA |
| 10657 | 2016 | GAU | ADULT | 33 | Y | Blood culture | Bacteraemia without focus | II | Positive | t012 | MRSA |
| 10621 | 2016 | GAU | PAED | 12 | D | Blood culture | Bacteraemia without focus | III | Positive | t037 | MRSA |
| 10722 | 2016 | GAU | ADULT | 50 | Y | Blood culture | Skin/soft tissue infection | SCCmec type V or SCCmec type VII | Positive | t045 | MRSA |
| 10806 | 2016 | GAU | PAED | 9 | Y | Blood culture | Bacteraemia without focus | IV | Positive | t718 | MRSA |
| 11188 | 2017 | GAU | PAED | 9 | D | Blood culture | Bacteraemia without focus | III | Positive | t037 | MRSA |
| 11384 | 2017 | GAU | PAED | 27 | D | Blood culture | Bacteraemia without focus | III | Positive | t037 | MRSA |
| 11469 | 2017 | GAU | ADULT | 26 | Y | Blood culture | Bacteraemia without focus | SCCmec type-I like (1,5B) | Positive | t045 | MRSA |
| 11472 | 2017 | GAU | ADULT | 34 | Y | Blood culture | Bacteraemia without focus | IV | Positive | t1257 | MRSA |
| 11484 | 2017 | GAU | ADULT | 65 | Y | Blood culture | Bacteraemia without focus | III | Positive | t037 | MRSA |
| 11487 | 2017 | GAU | PAED | 1 | M | Blood culture | Bacteraemia without focus | III | Positive | t037 | MRSA |
| 11518 | 2017 | GAU | ADULT | 62 | Y | Blood culture | LRTI | IV | Positive | t1257 | MRSA |
| 11522 | 2017 | GAU | ADULT | 81 | Y | Blood culture | Bacteraemia without focus | III | Positive | t037 | MRSA |
| 11528 | 2017 | GAU | PAED | 8 | D | Blood culture | Bacteraemia without focus | III | Positive | t037 | MRSA |
| 11578 | 2017 | GAU | PAED | 1 | M | Blood culture | Bacteraemia without focus | III | Positive | t037 | MRSA |
| 11688 | 2017 | GAU | PAED | 20 | D | Blood culture | Bacteraemia without focus | III | Positive | t037 | MRSA |
| 11706 | 2017 | GAU | Paed | 2 | M | Blood culture | - | III | Positive | t037 | MRSA |
| 12036 | 2017 | GAU | ADULT | 52 | Y | Blood culture | LRTI | II | Positive | t012 | MRSA |
| 12037 | 2017 | GAU | PAED | 12 | D | Blood culture | Bacteraemia without focus | SCCmec type-I like (1,5B) | Positive | t045 | MRSA |
| 11997 | 2017 | GAU | ADULT | 24 | Y | Blood culture | Bacteraemia without focus | II | Positive | t012 | MRSA |
| 12271 | 2017 | GAU | ADULT | 68 | Y | Blood culture | Skin/soft tissue infection | IV | Positive | t022 | MRSA |
| 12326 | 2017 | GAU | PAED | 8 | D | Blood culture | Bacteraemia without focus | III | Positive | t037 | MRSA |
| 12461 | 2017 | GAU | PAED | 5 | D | Blood culture | Bacteraemia without focus | New SCCmec type combination 5C | Positive | t037 | MRSA |
| 12459 | 2017 | GAU | PAED | 13 | D | Blood culture | Bacteraemia without focus | III | Positive | t5691 | MRSA |
| 12465 | 2017 | GAU | PAED | 0 | D | Blood culture | Bacteraemia without focus | III | Positive | t037 | MRSA |
| 12527 | 2017 | GAU | ADULT | 34 | Y | Blood culture | Bacteraemia without focus | II | Positive | t012 | MRSA |
| 12650 | 2017 | GAU | PAED | 2 | M | Blood culture | Bacteraemia without focus | SCCmec type-I like (1,5B) | Positive | t045 | MRSA |
| 12686 | 2017 | GAU | PAED | 16 | D | Blood culture | Other | III | Positive | t037 | MRSA |
| 12693 | 2017 | GAU | PAED | 11 | D | Blood culture | Bacteraemia without focus | III | Positive | t037 | MRSA |
| 12725 | 2017 | GAU | PAED | 8 | D | Blood culture | Bacteraemia without focus | III | Positive | t037 | MRSA |
| 12819 | 2017 | GAU | PAED | 14 | Y | Blood culture | Bacteraemia without focus | IV | Positive | t1257 | MRSA |
| 12820 | 2017 | GAU | ADULT | 47 | Y | Blood culture | Bacteraemia without focus | IV | Positive | t1257 | MRSA |
| 12821 | 2017 | GAU | PAED | 1 | M | Blood culture | Bacteraemia without focus | III | Positive | t19935 | MRSA |
| 7863 | 2014 | WC | ADULT | 84 | Y | Blood culture | LRTI | II | Positive | t012 | MRSA |
| 7880 | 2014 | WC | ADULT | 22 | Y | Blood culture | Bacteraemia without focus | II | Positive | t012 | MRSA |
| 8124 | 2014 | WC | ADULT | 50 | Y | Blood culture | LRTI | II | Positive | t012 | MRSA |
| 8158 | 2014 | WC | ADULT | 76 | Y | Blood culture | Bacteraemia without focus | II | Positive | t012 | MRSA |
| 8160 | 2014 | WC | ADULT | 42 | Y | Blood culture | Bacteraemia without focus | II | Positive | t012 | MRSA |
| 8307 | 2014 | WC | ADULT | 76 | Y | Blood culture | Bacteraemia without focus | II | Positive | t012 | MRSA |
| 8354 | 2014 | WC | ADULT | 16 | Y | Blood culture | Skin/soft tissue infection | SCCmec type III-like (3,5A) | Positive | t012 | MRSA |
| 8434 | 2014 | WC | ADULT | 62 | Y | Blood culture | Bacteraemia without focus | II | Positive | t012 | MRSA |
| 8463 | 2014 | WC | ADULT | 83 | Y | Blood culture | Bacteraemia without focus | II | Positive | t012 | MRSA |
| 8538 | 2014 | WC | ADULT | 58 | Y | Blood culture | Other | IV | Positive | t012 | MRSA |
| 8621 | 2014 | WC | ADULT | 64 | Y | Blood culture | Bacteraemia without focus | II | Positive | t012 | MRSA |
| 8697 | 2014 | WC | ADULT | 27 | Y | Blood culture | Bacteraemia without focus | II | Positive | t012 | MRSA |
| 8792 | 2014 | WC | ADULT | 29 | Y | Blood culture | Bacteraemia without focus | II | Positive | t012 | MRSA |
| 8838 | 2015 | WC | ADULT | 70 | Y | Blood culture | LRTI | II | Positive | t012 | MRSA |
| 9278 | 2015 | WC | ADULT | 64 | Y | Blood culture | Bacteraemia without focus | II | Positive | t012 | MRSA |
| 9554 | 2015 | WC | ADULT | 42 | Y | Blood culture | LRTI | II | Positive | t012 | MRSA |
| 9683 | 2015 | WC | ADULT | 67 | Y | Blood culture | LRTI | II | Positive | t012 | MRSA |
| 9953 | 2015 | WC | ADULT | 19 | Y | Blood culture | Bacteraemia without focus | II | Positive | t012 | MRSA |
| 9956 | 2015 | WC | ADULT | 36 | Y | Blood culture | Skin/soft tissue infection | II | Positive | t012 | MRSA |
| 9961 | 2015 | WC | ADULT | 62 | Y | Blood culture | Bacteraemia without focus | II | Positive | t012 | MRSA |
| 10442 | 2016 | WC | ADULT | 79 | Y | Blood culture | LRTI | II | Positive | t012 | MRSA |
| 10484 | 2016 | WC | ADULT | 22 | Y | Blood culture | Bacteraemia without focus | II | Positive | t012 | MRSA |
| 10548 | 2016 | WC | ADULT | 49 | Y | Blood culture | Bacteraemia without focus | II | Positive | t012 | MRSA |
| 10556 | 2016 | WC | ADULT | 39 | Y | Blood culture | Bacteraemia without focus | II | Positive | t012 | MRSA |
| 10906 | 2016 | WC | ADULT | 62 | Y | Blood culture | Bacteraemia without focus | II | Positive | t012 | MRSA |
| 11044 | 2016 | WC | ADULT | 45 | Y | Blood culture | LRTI | II | Positive | t012 | MRSA |
| 11057 | 2016 | WC | ADULT | 58 | Y | Blood culture | Bacteraemia without focus | II | Positive | t012 | MRSA |
| 11413 | 2017 | WC | ADULT | 22 | Y | Blood culture | Other | II | Positive | t012 | MRSA |
| 11416 | 2017 | WC | ADULT | 18 | Y | Blood culture | Skin/soft tissue infection | II | Positive | t012 | MRSA |
| 12049 | 2017 | WC | ADULT | 63 | Y | Blood culture | LRTI | IV | Positive | t012 | MRSA |
|  | 2017 | WC | ADULT | 58 | Y | Blood culture | Bacteraemia without focus | II | Positive | t012 | MRSA |
| 12596 | 2017 | WC | ADULT | 45 | Y | Blood culture | Bacteraemia without focus | II | Positive | t012 | MRSA |
| 12753 | 2017 | WC | ADULT | 69 | Y | Blood culture | LRTI | II | Positive | t012 | MRSA |
| 8925 | 2015 | WC | PAED | 19 | D | Blood culture | Bacteraemia without focus | II | Positive | t012 | MRSA |
| 11520 | 2017 | WC | PAED | 2 | M | Blood culture | LRTI | II | Positive | t012 | MRSA |
| 11607 | 2017 | WC | PAED | 27 | D | Blood culture | Bacteraemia without focus | II | Positive | t012 | MRSA |
| 11606 | 2017 | WC | PAED | 12 | D | Blood culture | Bacteraemia without focus | II | Positive | t012 | MRSA |
| 12544 | 2017 | WC | PAED | 38 | M | Blood culture | Other | II | Positive | t012 | MRSA |
| 11187 | 2016 | WC | ADULT | 66 | Y | Blood culture | Bacteraemia without focus | II | Positive | t0121 | MRSA |
| 8444 | 2014 | WC | PAED | 3 | M | Blood culture | Bacteraemia without focus | I | Positive | t015 | MRSA |
| 7839 | 2014 | WC | ADULT | 55 | Y | Blood culture | Bacteraemia without focus | II | Positive | t018 | MRSA |
| 9580 | 2015 | WC | ADULT | 53 | Y | Blood culture | Bacteraemia without focus | II | Positive | t021 | MRSA |
| 11292 | 2017 | WC | ADULT | 37 | Y | Blood culture | Bacteraemia without focus | II | Positive | t021 | MRSA |
| 8390 | 2014 | WC | ADULT | 60 | Y | Blood culture | LRTI | IV | Positive | t032 | MRSA |
| 8794 | 2014 | WC | ADULT | 60 | Y | Blood culture | Bacteraemia without focus | IV | Positive | t032 | MRSA |
| 9563 | 2015 | WC | ADULT | 57 | Y | Blood culture | Bacteraemia without focus | IV | Positive | t032 | MRSA |
| 9588 | 2015 | WC | ADULT | 18 | Y | Blood culture | Bacteraemia without focus | IV | Positive | t032 | MRSA |
| 9843 | 2015 | WC | ADULT | 59 | Y | Blood culture | Bacteraemia without focus | IV | Positive | t032 | MRSA |
| 10038 | 2015 | WC | ADULT | 65 | Y | Blood culture | Bacteraemia without focus | IV | Positive | t032 | MRSA |
| 10486 | 2016 | WC | ADULT | 24 | Y | Blood culture | Bacteraemia without focus | IV | Positive | t032 | MRSA |
| 10815 | 2016 | WC | ADULT | 63 | Y | Blood culture | Bacteraemia without focus | SCCmec type IV (2B) | Positive | t032 | MRSA |
| 11041 | 2016 | WC | ADULT | 30 | Y | Blood culture | Bacteraemia without focus | IV | Positive | t032 | MRSA |
| 11106 | 2016 | WC | ADULT | 48 | Y | Blood culture | Bacteraemia without focus | IV | Positive | t032 | MRSA |
| 11126 | 2017 | WC | ADULT | 51 | Y | Blood culture | Bacteraemia without focus | IV | Positive | t032 | MRSA |
| 11324 | 2017 | WC | ADULT | 77 | Y | Blood culture | Bacteraemia without focus | IV | Positive | t032 | MRSA |
| 12062 | 2017 | WC | ADULT | 82 | Y | Blood culture | Bacteraemia without focus | IV | Positive | t032 | MRSA |
| 12416 | 2017 | WC | ADULT | 57 | Y | Blood culture | Joint infection | IV | Positive | t032 | MRSA |
| 9210 | 2015 | WC | PAED | 6 | D | Blood culture | Bacteraemia without focus | IV | Positive | t032 | MRSA |
| 9270 | 2015 | WC | PAED | 2 | M | Blood culture | Bacteraemia without focus | IV | Positive | t032 | MRSA |
| 10648 | 2016 | WC | PAED | 9 | D | Blood culture | Bacteraemia without focus | SCCmec type-I like (1,5B) | Positive | t032 | MRSA |
| 7881 | 2014 | WC | ADULT | 50 | Y | Blood culture | Bacteraemia without focus | III | Positive | t037 | MRSA |
| 7934 | 2014 | WC | ADULT | 51 | Y | Blood culture | Skin/soft tissue infection | III | Positive | t037 | MRSA |
| 8225 | 2014 | WC | ADULT | 26 | Y | Blood culture | Skin/soft tissue infection | III | Positive | t037 | MRSA |
| 8281 | 2014 | WC | ADULT | 52 | Y | Blood culture | Skin/soft tissue infection | III | Positive | t037 | MRSA |
| 8310 | 2014 | WC | ADULT | 39 | Y | Blood culture | Bacteraemia without focus | III | Positive | t037 | MRSA |
| 8350 | 2014 | WC | ADULT | 52 | Y | Blood culture | Skin/soft tissue infection | III | Positive | t037 | MRSA |
| 8401 | 2014 | WC | ADULT | 72 | Y | Blood culture | LRTI | II | Positive | t037 | MRSA |
| 8459 | 2014 | WC | ADULT | 30 | Y | Blood culture | Skin/soft tissue infection | III | Positive | t037 | MRSA |
| 8500 | 2014 | WC | ADULT | 36 | Y | Blood culture | Skin/soft tissue infection | III | Positive | t037 | MRSA |
| 8531 | 2014 | WC | ADULT | 24 | Y | Blood culture | Skin/soft tissue infection | III | Positive | t037 | MRSA |
| 8539 | 2014 | WC | ADULT | 30 | Y | Blood culture | Skin/soft tissue infection | III | Positive | t037 | MRSA |
| 8568 | 2014 | WC | ADULT | 56 | Y | Blood culture | Bacteraemia without focus | III | Positive | t037 | MRSA |
| 8580 | 2014 | WC | ADULT | 31 | Y | Blood culture | Skin/soft tissue infection | III | Positive | t037 | MRSA |
| 8583 | 2014 | WC | ADULT | 28 | Y | Blood culture | Skin/soft tissue infection | III | Positive | t037 | MRSA |
| 8604 | 2014 | WC | ADULT | 17 | Y | Blood culture | Skin/soft tissue infection | III | Positive | t037 | MRSA |
| 8619 | 2014 | WC | ADULT | 29 | Y | Blood culture | Skin/soft tissue infection | III | Positive | t037 | MRSA |
| 8707 | 2014 | WC | ADULT | 32 | Y | Blood culture | Skin/soft tissue infection | III | Positive | t037 | MRSA |
| 8693 | 2014 | WC | ADULT | 68 | Y | Blood culture | Bacteraemia without focus | III | Positive | t037 | MRSA |
| 8745 | 2014 | WC | ADULT | 17 | Y | Blood culture | Skin/soft tissue infection | III | Positive | t037 | MRSA |
| 8804 | 2015 | WC | ADULT | 26 | Y | Blood culture | Bacteraemia without focus | III | Positive | t037 | MRSA |
| 9025 | 2015 | WC | ADULT | 67 | Y | Blood culture | Bacteraemia without focus | II | Positive | t037 | MRSA |
| 9334 | 2015 | WC | ADULT | 43 | Y | Blood culture | Bacteraemia without focus | III | Positive | t037 | MRSA |
| 9564 | 2015 | WC | ADULT | 31 | Y | Blood culture | Bacteraemia without focus | III | Positive | t037 | MRSA |
| 9821 | 2015 | WC | ADULT | 67 | Y | Blood culture | Bacteraemia without focus | III | Positive | t037 | MRSA |
| 10233 | 2016 | WC | ADULT | 34 | Y | Blood culture | Skin/soft tissue infection | III | Positive | t037 | MRSA |
| 10404 | 2016 | WC | ADULT | 37 | Y | Blood culture | Bacteraemia without focus | IV | Positive | t037 | MRSA |
| 10619 | 2016 | WC | ADULT | 35 | Y | Blood culture | Bacteraemia without focus | III | Positive | t037 | MRSA |
| 10643 | 2016 | WC | ADULT | 28 | Y | Blood culture | Bacteraemia without focus | III | Positive | t037 | MRSA |
| 10665 | 2016 | WC | ADULT | 66 | Y | Blood culture | Bacteraemia without focus | II | Positive | t037 | MRSA |
| 10884 | 2016 | WC | ADULT | 15 | Y | Blood culture | Bacteraemia without focus | III | Positive | t037 | MRSA |
| 10899 | 2016 | WC | ADULT | 26 | Y | Blood culture | Bacteraemia without focus | III | Positive | t037 | MRSA |
| 10901 | 2016 | WC | ADULT | 32 | Y | Blood culture | Bacteraemia without focus | III | Positive | t037 | MRSA |
| 10910 | 2016 | WC | ADULT | 37 | Y | Blood culture | Bacteraemia without focus | III | Positive | t037 | MRSA |
| 11040 | 2016 | WC | ADULT | 14 | Y | Blood culture | Bacteraemia without focus | III | Positive | t037 | MRSA |
| 11110 | 2016 | WC | ADULT | 34 | Y | Blood culture | Skin/soft tissue infection | III | Positive | t037 | MRSA |
| 11325 | 2017 | WC | ADULT | 48 | Y | Blood culture | Bacteraemia without focus | III | Positive | t037 | MRSA |
| 11540 | 2017 | WC | ADULT | 21 | Y | Blood culture | Bacteraemia without focus | III | Positive | t037 | MRSA |
| 12052 | 2017 | WC | ADULT | 27 | Y | Blood culture | Bacteraemia without focus | III | Positive | t037 | MRSA |
| 12053 | 2017 | WC | ADULT | 55 | Y | Blood culture | Bacteraemia without focus | III | Positive | t037 | MRSA |
| 12061 | 2017 | WC | ADULT | 29 | Y | Blood culture | Bacteraemia without focus | III | Positive | t037 | MRSA |
| 12082 | 2017 | WC | ADULT | 67 | Y | Blood culture | Bacteraemia without focus | III | Positive | t037 | MRSA |
| 12274 | 2017 | WC | ADULT | 30 | Y | Blood culture | Bacteraemia without focus | III | Positive | t037 | MRSA |
| 12381 | 2017 | WC | ADULT | 37 | Y | Blood culture | Bacteraemia without focus | III | Positive | t037 | MRSA |
| 13032 | 2017 | WC | ADULT | 60 | Y | Blood culture | Skin/soft tissue infection | III | Positive | t037 | MRSA |
| 12703 | 2017 | WC | ADULT | 47 | Y | Blood culture | Bacteraemia without focus | III | Positive | t037 | MRSA |
| 12765 | 2017 | WC | ADULT | 33 | Y | Blood culture | LRTI | III | Positive | t037 | MRSA |
| 12767 | 2017 | WC | ADULT | 66 | Y | Blood culture | LRTI | III | Positive | t037 | MRSA |
| 9488 | 2015 | WC | PAED | 9 | D | Blood culture | Bacteraemia without focus | SCCmec type I-like (1,5B) | Positive | t037 | MRSA |
| 9492 | 2015 | WC | PAED | 17 | D | Blood culture | Bacteraemia without focus | SCCmec type I-like (1,5B) | Positive | t037 | MRSA |
| 10727 | 2016 | WC | PAED | 10 | D | Blood culture | Bacteraemia without focus | SCCmec type-I like (1,5B) | Positive | t037 | MRSA |
| 10838 | 2016 | WC | PAED | 17 | D | Blood culture | Joint infection | SCCmec type-I like (1,5B) | Positive | t037 | MRSA |
| 10854 | 2016 | WC | PAED | 1 | M | Blood culture | Bacteraemia without focus | SCCmec typing inconclusive. Multiple ccr gene complexes. Polyclonal infection can not be excluded | Positive | t037 | MRSA |
| 11109 | 2016 | WC | PAED | 13 | Y | Blood culture | LRTI | III | Positive | t037 | MRSA |
| 12589 | 2017 | WC | PAED | 4 | M | Blood culture | Bacteraemia without focus | III | Positive | t037 | MRSA |
| 8366 | 2014 | WC | ADULT | 57 | Y | Blood culture | Bacteraemia without focus | III | Positive | t045 | MRSA |
| 9192 | 2015 | WC | ADULT | 35 | Y | Blood culture | Bacteraemia without focus | V | Positive | t045 | MRSA |
| 10717 | 2016 | WC | ADULT | 79 | Y | Blood culture | Skin/soft tissue infection | II | Positive | t045 | MRSA |
| 10851 | 2016 | WC | ADULT | 35 | Y | Blood culture | Bacteraemia without focus | III | Positive | t045 | MRSA |
| 12532 | 2017 | WC | ADULT | 63 | Y | Blood culture | Bacteraemia without focus | IV | Positive | t045 | MRSA |
| 7840 | 2014 | WC | PAED | 7 | D | Blood culture | Bacteraemia without focus | SCCmec type I-like (1,5B) | Positive | t045 | MRSA |
| 7843 | 2014 | WC | PAED | 7 | D | Blood culture | Bacteraemia without focus | SCCmec type I-like (1,5B) | Positive | t045 | MRSA |
| 7845 | 2014 | WC | PAED | 1 | D | Blood culture | Bacteraemia without focus | SCCmec type I-like (1,5B) | Positive | t045 | MRSA |
| 7862 | 2014 | WC | PAED | 13 | D | Blood culture | Bacteraemia without focus | SCCmec type I-like (1,5B) | Positive | t045 | MRSA |
| 8005 | 2014 | WC | PAED | 2 | D | Blood culture | Bacteraemia without focus | SCCmec type I-like (1,5B) | Positive | t045 | MRSA |
| 8231 | 2014 | WC | PAED | 12 | D | Blood culture | Bacteraemia without focus | SCCmec type I-like (1,5B) | Positive | t045 | MRSA |
| 8499 | 2014 | WC | PAED | 11 | D | Blood culture | Bacteraemia without focus | SCCmec type I-like (1,5B) | Positive | t045 | MRSA |
| 8585 | 2014 | WC | PAED | 6 | D | Blood culture | LRTI | SCCmec type I-like (1,5B) | Positive | t045 | MRSA |
| 8750 | 2014 | WC | PAED | 2 | M | Blood culture | Bacteraemia without focus | SCCmec type I-like (1,5B) | Positive | t045 | MRSA |
| 8837 | 2015 | WC | PAED | 19 | M | Blood culture | LRTI | SCCmec type I-like (1,5B) | Positive | t045 | MRSA |
| 9197 | 2015 | WC | PAED | 2 | M | Blood culture | Bacteraemia without focus | III | Positive | t045 | MRSA |
| 9280 | 2015 | WC | PAED | 3 | D | Blood culture | Bacteraemia without focus | mec class not identified, even though mecA positive | Positive | t045 | MRSA |
| 9335 | 2015 | WC | PAED | 28 | D | Blood culture | Bacteraemia without focus | V | Positive | t045 | MRSA |
| 9336 | 2015 | WC | PAED | 22 | D | Blood culture | LRTI | SCCmec type I-like (1,5B) | Positive | t045 | MRSA |
| 9560 | 2015 | WC | PAED | 3 | M | Blood culture | Bacteraemia without focus | SCCmec type I-like (1,5B) | Positive | t045 | MRSA |
| 9569 | 2015 | WC | PAED | 28 | D | Blood culture | Bacteraemia without focus | SCCmec type I-like (1,5B) | Positive | t045 | MRSA |
| 9957 | 2015 | WC | PAED | 11 | D | Blood culture | Bacteraemia without focus | SCCmec type I-like (1,5B) | Positive | t045 | MRSA |
| 9960 | 2015 | WC | PAED | 12 | D | Blood culture | Bacteraemia without focus | SCCmec type I-like (1,5B) | Positive | t045 | MRSA |
| 10000 | 2015 | WC | PAED | 2 | D | Blood culture | Bacteraemia without focus | SCCmec type I-like (1,5B) | Positive | t045 | MRSA |
| 10040 | 2015 | WC | Paed | 8 | D | Blood culture |  | SCCmec type I-like (1,5B) | Positive | t045 | MRSA |
| 10044 | 2015 | WC | PAED | 6 | D | Blood culture | Bacteraemia without focus | SCCmec type I-like (1,5B) | Positive | t045 | MRSA |
| 10053 | 2015 | WC | PAED | 6 | D | Blood culture | Skin/soft tissue infection | SCCmec type I-like (1,5B) | Positive | t045 | MRSA |
| 10319 | 2016 | WC | PAED | 3 | M | Blood culture | LRTI | Mec gene complex could not be amplified. Ccr gene complex 1 and 5 positive. Based on spa-type suggestive of SCCmec type I-like, but need to be confirmed with sequencing of complete SCCmec element | Positive | t045 | MRSA |
| 10443 | 2016 | WC | PAED | 1 | M | Blood culture | Bacteraemia without focus | SCCmec type-I like (1,5B) | Positive | t045 | MRSA |
| 10560 | 2016 | WC | PAED | 3 | M | Blood culture | LRTI | SCCmec type-I like (1,5B) | Positive | t045 | MRSA |
| 10587 | 2016 | WC | PAED | 7 | D | Blood culture | Bacteraemia without focus | SCCmec type-I like (1,5B) | Positive | t045 | MRSA |
| 10628 | 2016 | WC | PAED | 13 | D | Blood culture | Bacteraemia without focus | SCCmec type-I like (1,5B) | Positive | t045 | MRSA |
| 10834 | 2016 | WC | PAED | 5 | D | Blood culture | Bacteraemia without focus | SCCmec type-I like (1,5B) | Positive | t045 | MRSA |
| 10887 | 2016 | WC | PAED | 5 | D | Blood culture | LRTI | SCCmec type I-like (1,5B) | Positive | t045 | MRSA |
| 10920 | 2016 | WC | PAED | 6 | D | Blood culture | Abscess | SCCmec type-I like (1,5B) | Positive | t045 | MRSA |
| 10944 | 2016 | WC | PAED | 9 | D | Blood culture | Bacteraemia without focus | SCCmec type-I like (1,5B) | Positive | t045 | MRSA |
| 11039 | 2016 | WC | PAED | 7 | D | Blood culture | Bacteraemia without focus | SCCmec type-I like (1,5B) | Positive | t045 | MRSA |
| 11045 | 2016 | WC | PAED | 17 | D | Blood culture | LRTI | SCCmec type-I like (1,5B) | Positive | t045 | MRSA |
| 11162 | 2017 | WC | PAED | 4 | D | Blood culture | Bacteraemia without focus | SCCmec type-I like (1,5B) | Positive | t045 | MRSA |
| 11093 | 2016 | WC | PAED | 13 | D | Blood culture | Bacteraemia without focus | SCCmec type-I like (1,5B) | Positive | t045 | MRSA |
| 11149 | 2017 | WC | PAED | 5 | D | Blood culture | Bacteraemia without focus | SCCmec type-I like (1,5B) | Positive | t045 | MRSA |
| 11169 | 2017 | WC | PAED | 7 | D | Blood culture | Bacteraemia without focus | SCCmec type-I like (1,5B) | Positive | t045 | MRSA |
| 11192 | 2017 | WC | PAED | 5 | D | Blood culture | Bacteraemia without focus | III | Positive | t045 | MRSA |
| 11346 | 2017 | WC | PAED | 7 | D | Blood culture | Bacteraemia without focus | Mec gene complex could not be amplified. Ccr gene complex 1 and 5 positive. Based on spa-type suggestive of SCCmec type I-like, but need to be confirmed with sequencing of complete SCCmec element | Positive | t045 | MRSA |
| 11415 | 2017 | WC | PAED | 1 | M | Blood culture | Bacteraemia without focus | SCCmec type-I like (1,5B) | Positive | t045 | MRSA |
| 11517 | 2017 | WC | PAED | 11 | D | Blood culture | Bacteraemia without focus | SCCmec type-I like (1,5B) | Positive | t045 | MRSA |
| 11574 | 2017 | WC | PAED | 5 | D | Blood culture | LRTI | SCCmec type-I like (1,5B) | Positive | t045 | MRSA |
| 11608 | 2017 | WC | PAED | 18 | D | Blood culture | Joint infection | SCCmec type-I like (1,5B) | Positive | t045 | MRSA |
| 11605 | 2017 | WC | PAED | 7 | D | Blood culture | Bacteraemia without focus | SCCmec type-I like (1,5B) | Positive | t045 | MRSA |
| 11609 | 2017 | WC | PAED | 7 | D | Blood culture | Bacteraemia without focus | SCCmec type-I like (1,5B) | Positive | t045 | MRSA |
| 11692 | 2017 | WC | PAED | 7 | D | Blood culture | Bacteraemia without focus | SCCmec type-I like (1,5B) | Positive | t045 | MRSA |
| 12058 | 2017 | WC | PAED | 3 | D | Blood culture | Bacteraemia without focus | SCCmec type-I like (1,5B) | Positive | t045 | MRSA |
| 12966 | 2017 | WC | PAED | 1 | M | Blood culture | Bacteraemia without focus | SCCmec type-I like (1,5B) | Positive | t045 | MRSA |
| 12594 | 2017 | WC | PAED | 13 | D | Blood culture | LRTI | SCCmec type-I like (1,5B) | Positive | t045 | MRSA |
| 11486 | 2017 | WC | ADULT | 37 | Y | Blood culture | Bacteraemia without focus | SCCmec type V or SCCmec type VII | Positive | t11775 | MRSA |
| 8101 | 2014 | WC | ADULT | 29 | Y | Blood culture | Bacteraemia without focus | IV | Positive | t1257 | MRSA |
| 8304 | 2014 | WC | ADULT | 34 | Y | Blood culture | Bacteraemia without focus | IV | Positive | t1257 | MRSA |
| 8349 | 2014 | WC | ADULT | 32 | Y | Blood culture | Bacteraemia without focus | III | Positive | t1257 | MRSA |
| 8446 | 2014 | WC | ADULT | 50 | Y | Blood culture | LRTI | II | Positive | t1257 | MRSA |
| 8462 | 2014 | WC | ADULT | 37 | Y | Blood culture | Bacteraemia without focus | IV | Positive | t1257 | MRSA |
| 8571 | 2014 | WC | ADULT | 45 | Y | Blood culture | Bacteraemia without focus | IV | Positive | t1257 | MRSA |
| 8602 | 2014 | WC | ADULT | 32 | Y | Blood culture | LRTI | IV | Positive | t1257 | MRSA |
| 9298 | 2015 | WC | ADULT | 33 | Y | Blood culture | Bacteraemia without focus | IV | Positive | t1257 | MRSA |
| 9641 | 2015 | WC | Adult | 43 | Y | Blood culture | Bacteraemia without focus | IV | Positive | t1257 | MRSA |
| 9679 | 2015 | WC | ADULT | 38 | Y | Blood culture | Skin/soft tissue infection | IV | Positive | t1257 | MRSA |
| 9689 | 2015 | WC | ADULT | 23 | Y | Blood culture | Skin/soft tissue infection | IV | Positive | t1257 | MRSA |
| 9701 | 2015 | WC | ADULT | 27 | Y | Blood culture | Skin/soft tissue infection | IV | Positive | t1257 | MRSA |
| 9706 | 2015 | WC | ADULT | 36 | Y | Blood culture | Bacteraemia without focus | IV | Positive | t1257 | MRSA |
| 9943 | 2015 | WC | ADULT | 51 | Y | Blood culture | Bacteraemia without focus | IV | Positive | t1257 | MRSA |
| 9977 | 2015 | WC | ADULT | 31 | Y | Blood culture | Bacteraemia without focus | IV | Positive | t1257 | MRSA |
| 10216 | 2016 | WC | ADULT | 50 | Y | Blood culture | Skin/soft tissue infection | IV | Positive | t1257 | MRSA |
| 10440 | 2016 | WC | ADULT | 66 | Y | Blood culture | LRTI | IV | Positive | t1257 | MRSA |
| 10385 | 2016 | WC | ADULT | 24 | Y | Blood culture | Other | IV | Positive | t1257 | MRSA |
| 10689 | 2016 | WC | ADULT | 84 | Y | Blood culture | Bacteraemia without focus | IV | Positive | t1257 | MRSA |
| 10837 | 2016 | WC | ADULT | 43 | Y | Blood culture | Bacteraemia without focus | IV | Positive | t1257 | MRSA |
| 11036 | 2016 | WC | ADULT | 29 | Y | Blood culture | LRTI | IV | Positive | t1257 | MRSA |
| 11111 | 2017 | WC | ADULT | 33 | Y | Blood culture | Bacteraemia without focus | IV | Positive | t1257 | MRSA |
| 11127 | 2017 | WC | ADULT | 30 | Y | Blood culture | Bacteraemia without focus | IV | Positive | t1257 | MRSA |
| 12054 | 2017 | WC | ADULT | 34 | Y | Blood culture | Bacteraemia without focus | IV | Positive | t1257 | MRSA |
| 12446 | 2017 | WC | ADULT | 29 | Y | Blood culture | LRTI | IV | Positive | t1257 | MRSA |
| 8846 | 2015 | WC | PAED | 26 | D | Blood culture | Bacteraemia without focus | IV | Positive | t1257 | MRSA |
| 9509 | 2015 | WC | PAED | 9 | D | Blood culture | Bacteraemia without focus | SCCmec type I-like complex (1,5 B) | Positive | t1257 | MRSA |
| 9941 | 2015 | WC | PAED | 4 | M | Blood culture | LRTI | IV | Positive | t1257 | MRSA |
| 10770 | 2016 | WC | PAED | 27 | D | Blood culture | Bacteraemia without focus | SCCmec type-I like (1,5B) | Positive | t1257 | MRSA |
| 8866 | 2015 | WC | ADULT | 78 | Y | Blood culture | Joint infection | IV | Positive | t1467 | MRSA |
| 10720 | 2016 | WC | PAED | 0 | D | Blood culture | Bacteraemia without focus | SCCmec type-I like (1,5B) | Positive | t1476 | MRSA |
| 10223 | 2016 | WC | ADULT | 55 | Y | Blood culture | Bacteraemia without focus | IV | Positive | t1971 | MRSA |
| 10284 | 2016 | WC | ADULT | 58 | Y | Blood culture | LRTI | IV | Positive | t1971 | MRSA |
| 11515 | 2017 | WC | ADULT | 66 | Y | Blood culture | Bacteraemia without focus | IV | Positive | t1971 | MRSA |
| 8152 | 2014 | WC | PAED | 5 | M | Blood culture | LRTI | IV | Positive | t1971 | MRSA |
| 8903 | 2015 | WC | ADULT | 38 | Y | Blood culture | Bacteraemia without focus | VI | Positive | t223 | MRSA |
| 10306 | 2016 | WC | ADULT | 19 | Y | Blood culture | Bacteraemia without focus | SCCmec type V or SCCmec type VII | Positive | t2409 | MRSA |
| 9504 | 2015 | WC | ADULT | 25 | Y | Blood culture | LRTI | II | Positive | t2526 | MRSA |
| 11758 | 2017 | WC | ADULT | 91 | Y | Blood culture | LRTI | IV | Positive | t294 | MRSA |
| 8050 | 2014 | WC | Adult | 77 | Y | Blood culture | Bacteraemia without focus | IV | Positive | t304 | MRSA |
| 12419 | 2017 | WC | ADULT | 74 | Y | Blood culture | Bacteraemia without focus | IV | Positive | t324 | MRSA |
| 12417 | 2017 | WC | ADULT | 52 | Y | Blood culture | Skin/soft tissue infection | IV | Positive | t432 | MRSA |
| 9030 | 2015 | WC | PAED | 2 | D | Blood culture | Bacteraemia without focus | mec class not identified, even though mecA positive | Positive | t498 | MRSA |
| 8320 | 2014 | WC | PAED | 5 | Y | Blood culture | Bacteraemia without focus | SCCmec type V or VII | Positive | t5483 | MRSA |
| 12421 | 2017 | WC | ADULT | 37 | Y | Blood culture | LRTI | IV | Positive | t5691 | MRSA |
| 10335 | 2016 | WC | ADULT | 47 | Y | Blood culture | Bacteraemia without focus | IV | Positive | t578 | MRSA |
| 10476 | 2016 | WC | PAED | 8 | M | Blood culture | Bacteraemia without focus | II | Positive | t6330 | MRSA |
| 12340 | 2017 | WC | ADULT | 28 | Y | Blood culture | Bacteraemia without focus | SCCmec type-I like (1,5B) | Positive | t6931 | MRSA |
| 10814 | 2016 | WC | ADULT | 44 | Y | Blood culture | LRTI | IV | Positive | t718 | MRSA |
| 8321 | 2014 | WC | ADULT | 63 | Y | Blood culture | Bacteraemia without focus | III | Positive | t037 | MRSA |
| 9337 | 2015 | WC | ADULT | 23 | Y | Blood culture | Skin/soft tissue infection | II | Positive | t012 | MRSA |
| 9876 | 2015 | WC | ADULT | 89 | Y | Blood culture | Bacteraemia without focus | IV | Positive | t032 | MRSA |
| 8233 | 2014 | WC | PAED | 6 | D | Blood culture | Bacteraemia without focus | SCCmec type I-like (1,5B) | Positive | t045 | MRSA |
| 8062 | 2014 | WC | ADULT | 19 | Y | Blood culture | Bacteraemia without focus | II | Positive | t012 | MRSA |
| 8230 | 2014 | WC | ADULT | 44 | Y | Blood culture | LRTI | IV | Positive | t032 | MRSA |
| 8501 | 2014 | WC | ADULT | 68 | Y | Blood culture | Bacteraemia without focus | II | Positive | t012 | MRSA |
| 8129 | 2014 | WC | ADULT | 46 | Y | Blood culture | LRTI | IV | Positive | t718 | MRSA |
| 10903 | 2016 | WC | ADULT | 24 | Y | Blood culture | Bone infection | IV | Positive | t18226 | MRSA |
| 11049 | 2016 | WC | ADULT | 57 | Y | Blood culture | Bacteraemia without focus | IV | Positive | Untypeable | MRSA |
| 12752 | 2017 | WC | PAEDS | 13 | D | Blood culture | Bacteraemia without focus | IV | Positive | t1971 | MRSA |
| 10668 | 2016 | WC | ADULT | 53 | Y | Blood culture | Bacteraemia without focus | SCCmec type V or SCCmec type VII | Positive | t018 | MRSA |
| 12702 | 2017 | WC | ADULT | 46 | Y | Blood culture | Bacteraemia without focus | III | Positive | t037 | MRSA |
| 9299 | 2015 | WC | ADULT | 42 | Y | Blood culture | Bacteraemia without focus | SCCmec V or SCCmec VII (5C) | Positive | t1476 | MRSA |
| 10568 | 2016 | WC | ADULT | 21 | Y | Blood culture | LRTI | SCCmec type V or SCCmec type VII | Positive | t1476 | MRSA |
| 7853 | 2014 | WC | ADULT | 49 | Y | Blood culture | Bacteraemia without focus | II | Positive | t238 | MRSA |

GAU= Gauteng; WC= Western Cape; LRTI= Lower respiratory tract infection; MRSA= Methicillin-resistant *Staphylococcus aureus*
